# Bioavailability Enhancement of Poorly Water-Soluble Drugs via Nanocomposites: Formulation–Processing Aspects and Challenges

**DOI:** 10.3390/pharmaceutics10030086

**Published:** 2018-07-08

**Authors:** Anagha Bhakay, Mahbubur Rahman, Rajesh N. Dave, Ecevit Bilgili

**Affiliations:** Otto H. York Department of Chemical and Materials Engineering, New Jersey Institute of Technology, Newark, NJ 07102, USA; aab22@njit.edu (A.B.); mr485@njit.edu (M.R.); dave@njit.edu (R.N.D.)

**Keywords:** BCS Class II drugs, drug nanosuspensions, nanocomposites, redispersion, dissolution enhancement, aggregates, formulation

## Abstract

Drug nanoparticles embedded in a dispersant matrix as a secondary phase, i.e., drug-laden nanocomposites, offer a versatile delivery platform for enhancing the dissolution rate and bioavailability of poorly water-soluble drugs. Drug nanoparticles are prepared by top-down, bottom-up, or combinative approaches in the form of nanosuspensions, which are subsequently dried to prepare drug-laden nanocomposites. In this comprehensive review paper, the term “nanocomposites” is used in a broad context to cover drug nanoparticle-laden intermediate products in the form of powders, cakes, and extrudates, which can be incorporated into final oral solid dosages via standard pharmaceutical unit operations, as well as drug nanoparticle-laden strip films. The objective of this paper is to review studies from 2012–2017 in the field of drug-laden nanocomposites. After a brief overview of the various approaches used for preparing drug nanoparticles, the review covers drying processes and dispersant formulations used for the production of drug-laden nanocomposites, as well as various characterization methods including quiescent and agitated redispersion tests. Traditional dispersants such as soluble polymers, surfactants, other water-soluble dispersants, and water-insoluble dispersants, as well as novel dispersants such as wet-milled superdisintegrants, are covered. They exhibit various functionalities such as drug nanoparticle stabilization, mitigation of aggregation, formation of nanocomposite matrix–film, wettability enhancement, and matrix erosion/disintegration. Major challenges such as nanoparticle aggregation and poor redispersibility that cause inferior dissolution performance of the drug-laden nanocomposites are highlighted. Literature data are analyzed in terms of usage frequency of various drying processes and dispersant classes. We provide some engineering considerations in comparing drying processes, which could account for some of the diverging trends in academia vs. industrial practice. Overall, this review provides rationale and guidance for drying process selection and robust nanocomposite formulation development, with insights into the roles of various classes of dispersants.

## 1. Introduction

The number of newly developed drug molecules with greater lipophilicity, higher molecular weight, and poor water solubility has increased over the last few decades due to the emerging trends in combinatorial chemistry and drug design [1,2,3]. About 40% of drugs with market approval and nearly 90% of molecules in the discovery pipeline are poorly water-soluble [4]. The majority of failures in new drug development have been attributed to poor water solubility of the drug. It is well-known that poor solubility and slow dissolution can lead to low bioavailability, resulting in suboptimal drug delivery [5,6]. Commonly used approaches for enhancing the dissolution rate of these molecules include nanoparticle-based formulations [7,8], lipid-based drug delivery systems [9,10], pro-drugs [11,12], amorphous solid dispersions [13,14], salt formation [15,16], co-crystals [17,18], and cyclodextrin complexes [19,20].

Among the several approaches mentioned above, preparation of drug nanoparticles has been shown to be successful for improving the dissolution rate of a multitude of drugs, and 16 drugs have been marketed using drug nanoparticles (see Table 1). Drug nanoparticles have larger specific surface area and higher overall solute transfer coefficient than their micron-sized counterparts [21,22,23]. Moreover, ultrafine particles, especially those with sizes less than ~100 nm, tend to show higher saturation solubility, which can be explained via the Ostwald–Freundlich equation [24]. Overall, all these features exhibited by nanoparticles improve the dissolution rates according to the Noyes–Whiney equation [25]; this in turn enhances bioavailability [26,27]. Besides enhanced solubility and dissolution rates leading to improved bioavailability, other advantages of drug nanoparticles include the elimination of food effects, safe dose escalation, and enhanced efficacy and tolerability profiles [28,29,30].

Drug nanoparticles can be prepared in the form of suspensions, referred to as nanosuspensions, by top-down, bottom-up, or combinative methods. Top-down methods such as high-pressure homogenization (HPH) [32], stirred media milling [21,33,34], and ball milling [35] involve high shear–impact forces to achieve size reduction of coarse, as-received drug crystals down to micro or nanometer scale. Bottom-up methods involve building up particles by precipitation of dissolved molecules via liquid antisolvent precipitation (LASP) [36] and precipitation by supercritical fluids [37,38]. Melt emulsification is another example of bottom-up technique which can be used for drugs with low melting points. In this method, the drug is dispersed in an aqueous stabilizer solution and heated to melt crystals, followed by flash cooling to produce drug nanosuspensions [39,40]. Combinative methods [41] include a combination of bottom-up and top-down approaches.

Drug nanosuspensions must be physically stable during processing and storage for proper downstream processing or adequate shelf-life, depending on the intended final dosage form [23,28]. Important benefits resulting from high surface area can be lost if nanoparticles grow and/or form large clusters (aggregates). Moreover, suspensions that exhibit severe aggregation can pose significant downstream processing challenges due to high zero-shear viscosity and/or yield stress. As compared with microparticles, nanoparticles in a suspension show a strong tendency to aggregate because they have a high number concentration (given solid loading), small interparticle distances, enhanced Brownian motion, and relatively high surface energy [33,42,43,44]. During the preparation of drug nanosuspensions, turbulent mixing and high shear can cause faster aggregation if the nanosuspension is not properly stabilized upon use of various stabilizers [45,46]. The Brownian motion of nanoparticles may also contribute to the high collision rates during processing [44,47]. During the storage of the nanosuspensions, the Brownian motion is the major driving force for nanoparticle collisions besides gravity. Once the nanoparticles collide, they can aggregate due to van der Waals or hydrophobic forces, depending on their surface charge, which is quantified by zeta potential [48]. According to Müller [49], a zeta potential value of at least ±30 mV is required for an electrostatically stabilized suspension. About ±20 mV provides only a short-term stability, and values in the range of −5 mV to +5 mV indicate fast aggregation [50]. In the case of combined electrostatic and steric (electrosteric) stabilization, a minimum zeta potential of ±20 mV is desirable [51].

Stabilization of drug nanosuspensions can be achieved by electrostatic, steric, and electrosteric interactions of nanoparticle surfaces with adsorbing polymers and surfactants [48,52], also known as stabilizers. Most poorly water-soluble drugs exhibit hydrophobic behavior and cannot be well-dispersed in aqueous media without the addition of stabilizers, which also serve as wetting agents. On the other hand, many BCS Class II drugs exhibit finite solubility in the dispersion medium, which can be enhanced by the addition of stabilizers, especially surfactants. With increasing solubility, the particles may grow due to Ostwald ripening [53,54] especially if the suspensions are stored for a long time before down-stream processing such as filtration and drying.

**Figure 1:**
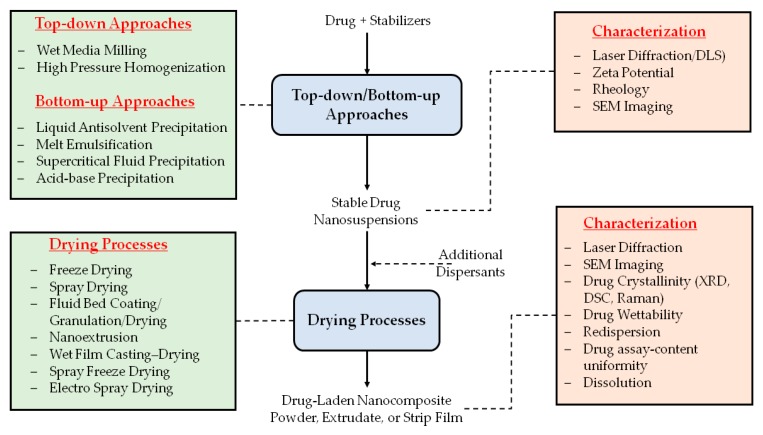
Schematic illustrating the steps involved in the preparation of drug-laden nanocomposites including their characterization. Nanocomposites in the form of powders, cakes, or extrudates are intermediate products that are incorporated into final solid oral dosage forms such as tablets, capsules, and sachets via standard pharmaceutical unit operations upon use of additional excipients. Polymeric strip films prepared by wet film casting–drying are the final product.

While drug nanosuspensions can be used as oral suspensions and injectables, most marketed products are developed as oral solid dosage forms (see Table 1), because the latter are preferred by patients and doctors for their relative ease of administration, accurate dosing, and stability [28,55]. Moreover, despite the use of stabilizers, it is challenging to ensure the long-term physical stability of drug nanosuspensions. In fact, drying is generally perceived as a stabilization step for nanocrystals to avoid typical deterioration occurring in a liquid nanosuspension, such as Ostwald ripening, particle aggregation, sedimentation, and creaming [56,57]. For all the aforementioned reasons, drug nanosuspensions have been dried, as illustrated in Figure 1, via spray drying [33,34,58,59,60,61,62], fluid bed coating/granulation/drying [8,60,63,64,65,66], spray-freeze drying [7,67], freeze drying [68,69,70,71,72], vacuum drying [73,74], nanoextrusion [75,76,77,78], and wet casting–drying [40,79,80,81,82,83]. Drying processes convert drug nanosuspensions into nanocomposites that encapsulate or carry drug nanoparticles and their clusters dispersed as a secondary phase in the matrix of dispersants (stabilizers used in nanosuspensions and other excipients). Depending on the drying method, nanocomposites can be in the form of powders, cakes, or extrudates, which can be integrated into tablets, capsules, and sachets via standard pharmaceutical unit operations. Alternatively, they are in the form of polymeric strip films.

**Figure 2:**
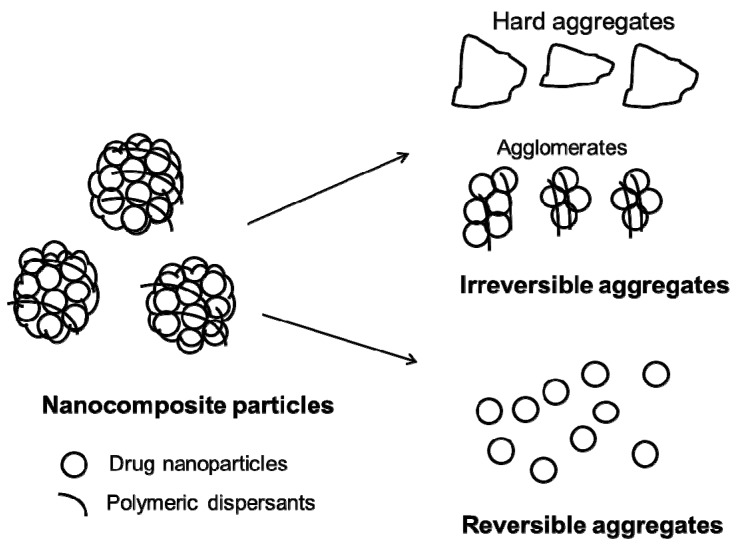
Classification of various types of aggregates that may be present in nanocomposite particles based on their redispersion behavior.

A major formulation challenge in dissolution enhancement upon use of drug-laden nanocomposites is that drug nanoparticles in nanocomposites may be released too slowly and/or in the form of large clusters (a.k.a. aggregates) during in vivo or vitro dissolution [66,84,85,86,87]. Besides the aggregation that may take place during the preparation/storage of drug nanosuspensions, drug nanoparticles can also aggregate into larger sub-micron clusters or even micron-sized clusters during the removal of water or solvents in the drying process, depending on the type/concentration of the dispersants [74,85]. Consequently, the advantages of drug nanoparticles with inherently large surface areas could be lost upon drying. The aggregates may be broadly classified as irreversible and reversible, as shown in Figure 2 [66,74,86], based on the redispersion behavior of dried nanosuspensions (nanocomposites) in liquids. Nanocomposite particles may contain aggregates of drug nanoparticles that have formed during the nanosuspension preparation step and/or drying step. Hard aggregates can be formed by the fusion of drug nanoparticles due to the removal of water/solvents during drying, especially when the dispersant concentration in the nanosuspension is too low (see e.g., [86]). They are most likely held together by solid bridges formed upon re-crystallization of some dissolved drug during drying. Agglomerates, another type of irreversible aggregates, could also form during drying. Although the exact mechanism leading to nanoparticle agglomeration is unknown [34], the capillary pressure theory is one theory that explains agglomeration due to the capillary forces encountered during the drying process [88]; others attributed agglomeration to polymer chain entanglement and/or potential micro-phase separation of polymeric stabilizer–other dispersants from particles upon increase in particle concentration with reduced water content [74,89,90]. Since irreversible aggregates do not redisperse back to primary nanoparticles, significant loss of drug surface area occurs, leading to inferior dissolution rate enhancement [66,85,91,92]. Unless otherwise indicated, aggregates in nanocomposites refer to irreversible aggregates in this paper.

A relevant concern is that even the reversible aggregates and primary drug nanoparticles in nanocomposites may be released too slowly from the dispersant matrix in aqueous media, which will lead to inferior dissolution rate and bioavailability. Not only does the dispersants’ type/concentration in the nanocomposite matrix affect the aggregation of drug nanoparticles in the nanosuspensions and dried nanocomposites, it also modulates the release of drug nanoparticles and their clusters, thus controlling the overall drug release rate [56,84]. Therefore, an understanding of nanoparticle recovery from nanocomposites after suspending nanocomposites in water (redispersion phenomenon) and its impact on drug dissolution rate is critically important [55,66,84,87,92]. The study of redispersion also sheds light on the functionalities of different classes of dispersants used in nanocomposites.

**Figure 3:**
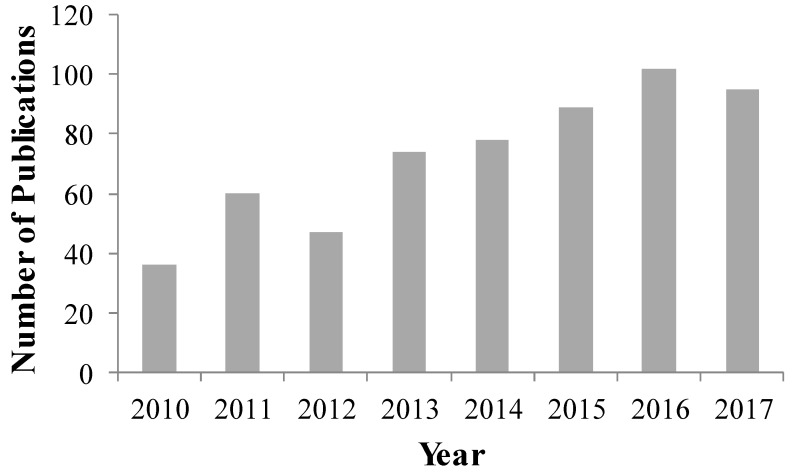
The number of published journal articles from 2010–2017 which reported the preparation of drug nanoparticles and drug nanocomposites. Source: Scopus database, key words used: “drug nanoparticles” or “drug nanocomposites” or “drug + drying + nanocrystals” or “drug + drying + nanosuspensions”.

About 600 publications are available in the Scopus database on drug nanoparticles and drug nanocomposites, with growing interest over the past 7 years (Figure 3). Several excellent review papers are available on this topic. Chin et al. [55] reviewed formulations and processes used for converting drug nanosuspensions to final drug products in publications up to 2012, as well as providing a review of patents. Brough and Williams [93] provided a comparative analysis of amorphous solid dispersions and nanocrystal technologies for poorly water-soluble drugs for oral delivery. Junghanns and Muller [29] summarized the approaches used to formulate the currently marketed products containing poorly water-soluble drugs. Kesisoglou et al. [28] described the principles of nanosizing, production, and characterization of nanoformulations, and in vivo impact of these formulations. Chogale et al. [94] mainly described characterization techniques for various characteristics of nanocrystals (particle size, saturation solubility, dissolution velocity), which have an impact on the improved performance of nanocrystals. Peltonen and Hirvonen [95] presented the most important properties of nanocrystalline drug compounds, with multiple examples of the development and characterization of nanocrystalline drug formulations and a focus on the role of higher saturation solubility. They explained the impact of polymers and surfactants on the stabilization of nanocrystals, with a few examples from the literature and marketed products, but did not do an in-depth analysis on the roles of various dispersants. Malamatari et al. [31] outlines the advantages, stabilization, and production of drug nanocrystals, with an emphasis on wet milling, while highlighting their pharmaceutical applications. Although there is some overlap among the aforementioned review papers, each one has a unique focus and different duration of literature covered. In general, most reviews neither highlighted the critical role of redispersion on dissolution rate improvement of poorly water-soluble drugs, nor did they discuss recently developed redispersion methods in detail. Unlike some of the previous reviews, we provide here a systematic critical analysis of different classes of dispersants in terms of their functionalities and impact on the aggregation–redispersion. As the most comprehensive review paper on nanosuspension drying, this paper provides a statistical analysis of the usage of various preparation methods for drug nanosuspensions and nanocomposites from 92 studies from 2012–2017. This review also covers novel drying methods such as nanoextrusion and wet film-casting–drying, as well as novel dispersants, which are largely missing from the previous reviews. Finally, it provides significant guidance and insight into the rational selection of a drying process and dispersant, in order to develop robust, redispersible, fast-dissolving nanocomposite formulations.

The organization of this review paper closely follows the sequence of preparation steps in Figure 1. Section 2 presents a short review of various approaches used for the preparation of drug nanosuspensions; the characterization of nanosuspensions is not discussed at length, as several review papers cover this topic in detail; readers are referred to these review papers and the references cited therein [23,55,94]. Section 3 presents a comprehensive review of various drying methods, methods for characterizing the drug-laden nanocomposites including the newly developed redispersion test methods, and formulation aspects such as functionalities of dispersants and their impact on redispersion/drug dissolution. It will also present a statistical analysis of the usage frequency of various preparation methods for drug nanoparticles–nanocomposites and various dispersant classes. Section 4 will present important engineering considerations that must be taken into account for the selection of a drying process, and explain some of the diverging trends between academic studies and industrial practice. Section 5 will provide various insights gained from the analysis of the data presented in Section 3, as well as practical guidance for the rational selection of dispersants for robust, streamlined formulation development of redispersible, fast-dissolving drug nanocomposites. Finally, specific applications of drug nanosuspensions and drug-laden nanocomposites in drug delivery, as well as patent landscape, have been extensively covered in previous reviews (e.g., [31,55]); hence, they are outside the scope of this review paper.

## 2. Preparation of Drug Nanosuspensions and Their Stabilization

### 2.1. Preparation Methods

**Figure 4:**
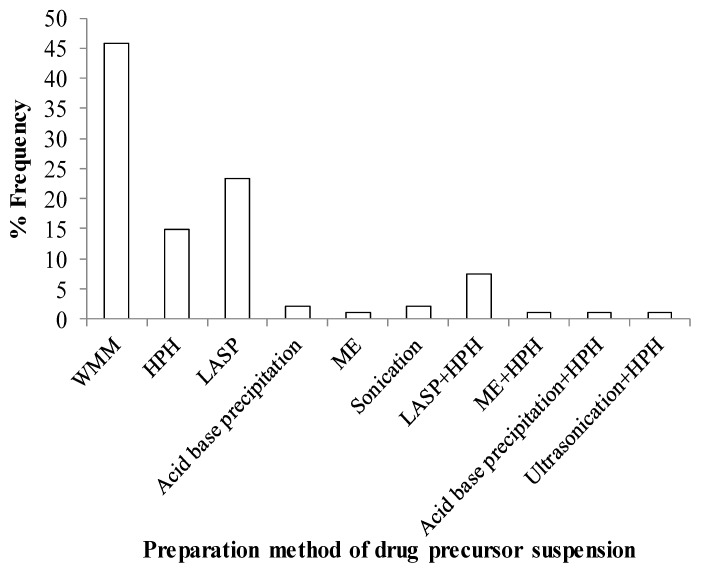
The usage frequency of various drug nanosuspension preparation methods in the studies reported in Table 2. The sample size for the analysis here is 94, even though the number of publications in Table 2 is 92, because two studies compared two different preparation methods.

Various methods used for the preparation of drug nanosuspensions in 2012–2017 studies are summarized in Table 2. Top-down approaches aim to break micron-sized drug crystals down to smaller micro or nanoparticles via shear–impact. Wet media milling (WMM) and high-pressure homogenization (HPH) are the most commonly used top-down approaches for particle size reduction (Figure 4). WMM is an organic solvent-free process that has several distinct advantages, such as production of suspensions with high drug loading, ability to run continuously, and good scalability. Moreover, it can be universally applied to most drug candidates with poor water-solubility [21,23,96]. In WMM, drug suspensions are prepared by dispersing drug particles in a stabilizer solution followed by particle size reduction in a media mill, where coarse drug particles are broken down into smaller particles by bead–bead collisions. Particle size during milling generally depends on process–equipment parameters, mechanical and physicochemical properties of drug particles, and the physical stability of the milled suspension, i.e., extent of aggregation and/or Ostwald ripening in the presence of various stabilizers [23,97,98]. Li et al. [23] provided a holistic view of various formulation–processing aspects of WMM, and concluded that preparation of a drug nanosuspension with desired particle size and adequate storage stability entails selecting a proper stabilizer formulation and effective process–equipment parameters for the WMM process. While either a polymer or a surfactant alone can be used for stabilization, a combination of a cellulosic polymer and an anionic surfactant has been shown to be effective in stabilizing multiple drug nanosuspensions [99,100,101]. The impact of bead size–loading, rotor speed, and drug loading on breakage kinetics, drug particle size, and operational efficiency was studied extensively via experimentation and microhydrodynamic models [102,103,104].

High-pressure homogenization (HPH) is another popular top-down method to produce drug nanoparticles [105]. It uses jet-stream homogenization by pumping drug, dispersion medium, and stabilizers through a micro fluidizing nozzle. The particle size reduction is caused by cavitation forces, shear forces, and collisions through multiple homogenization cycles. The process parameters that control the particle size are homogenization pressure, number of passes, drug loading, and stabilizer type–loading. Shen et al. [106] and Sun et al. [107] used HPC to produce stable drug nanosuspensions with a combination of steric and electrostatic stabilizers. Compared to WMM, HPH has the advantage of reduced product contamination due to wear because it does not use milling media.

Liquid antisolvent precipitation (LASP) [108,109], supercritical fluid precipitation [37,38], acid–base precipitation [110,111], and melt emulsification [39,40] are some of the common bottom-up methods. Here, we briefly describe LASP, as it is the most widely used bottom-up method (Figure 4), and melt emulsification, as it is a facile, cheap, and solvent-free bottom-up method. Particle formation by LASP involves the mixing of solution–antisolvent streams to generate supersaturation and fast precipitation of particles [36,69]. Uniform mixing conditions ensure rapid and uniform supersaturation, making it a precipitation-controlled process which results in the precipitation of ultra-fine particles with narrow particle-size distribution. However, the residual solvent should be quickly removed from the resulting suspension [112]; otherwise, it could lead to severe aggregation and particle growth [62,109]. Stabilizer screening involves selecting a favorable solvent and antisolvent system and stabilizers that can adsorb on the crystal surface as they form, thereby inhibiting crystal growth. Despite the simplicity of its design, low energy consumption, and absence of product contamination without any moving parts like in wet media milling, the LASP process has many challenges, such as residual solvents in suspensions, inadequate physical stability, and low drug loading in the suspensions [23,62,109].

Melt emulsification (ME) is a facile bottom-up method for preparing drug nanosuspensions [39,40]. In this process, an aqueous suspension of drug particles is heated to temperatures above the melting point of the drug to form an oil-in-water emulsion owing to the immiscibility of the molten drug–water. The hot emulsion is broken into smaller droplets by applying mechanical agitation via ultrasonication, homogenization, magnetic stirring, etc. Subsequent cooling of the emulsion leads to solidification–recrystallization of the drug droplets into nanoparticles. Obviously, ME is only applicable to drugs with melting points below the boiling point of water. The process parameters that affect particle size include sonication energy, cooling rate, drug loading, and suitable stabilizer selection. Knieke et al. [39] screened stabilizers using the hydrophilic–lipophilic balance concept to produce 30 wt.% fenofibrate nanosuspensions. They were able to produce nanosuspensions with smallest size of 150 nm using poloxamer 188 as the stabilizer by optimizing sonication energy, speed, drug loading, and stabilizer loading; however, the suspensions were physically stable at that size only for few min, thus requiring immediate solidification of the nanosuspension via drying.

Combinative methods have been claimed to increase efficiency of particle size reduction [41]. In general, they can be described as a combination of a bottom-up method followed by a top-down method such as LASP–HPH and ME–HPH. For example, Fu et al. [113] combined LASP and HPH to prepare nanosuspensions of Nimodipine. Nimodipine was dissolved in dimethyl sulfoxide (DMSO) and instantaneously precipitated in aqueous phase containing poloxamer 127, HPMC E5, and sodium deoxycholate. DMSO was removed by lyophilization to improve the stability of the nanosuspensions. If DMSO was not removed quickly enough, the drug particles would have grown. Apparently, combinative methods have not yet attracted as much attention as either WMM or LASP alone (Figure 4).

The suspensions prepared by bottom-up approaches typically have low drug loading and poor physical stability. The solvent used in LASP must be removed quickly, either by filtration–drying [109] or continuous drying [62,112], which entails more processing steps or elaborate process design–integration, respectively. In contrast, top-down methods, especially wet stirred media milling, offer significant advantages: they are considered more universal, i.e., applicability to a large class of BCS Class II drugs because of their capability of achieving high drug loading, organic solvent-free processing, continuous operation capability, and ease of scale up [22,23,31,98]. While wet stirred media milling is energy intensive and more costly and may cause unacceptable media wear/product contamination, recent investigations [102,103,104] and wide industrial practice exemplified in the manufacture of a multitude of marketed drug products (refer to Table 1) suggest that these issues can be easily mitigated by the judicious choice of process–equipment parameters. Hence, it is not surprising to see from Figure 4 that WMM was the most popular method of drug nanosuspension production in 2012–2017 studies. Similarly, it is the most widely used method for drug nanoparticle production in the marketed products (refer to Table 1). This convergence of published academic/industrial research and actual industry practice indeed suggests that WMM is the preferred method for pharmaceutical drug nanoparticle production.

### 2.2. More on the Stabilization of Drug Nanosuspensions

Since there are several review papers on the stabilization of drug nanosuspensions (e.g., [23,28,114]), this section will provide a brief review of the topic only. Electrostatic forces, steric forces, entropic forces, and van der Waals forces among nanoparticles determine the overall physical stability of a drug nanosuspension [57]. Stabilization of nanoparticles can be achieved by soluble polymers and/or surfactants as a class of dispersants known as stabilizers [56,115,116]. In fact, most of the soluble polymers and surfactants presented in Table 2 serve as stabilizers in the preparation of drug nanosuspensions. As mentioned in the Introduction, zeta potential of drug nanosuspensions is important to their stability. According to Müller [49], a zeta potential value of at least ±30 mV is required for an electrostatically stabilized suspension. About ±20 mV provides only a short-term stability, and values in the range −5 mV to +5 mV indicate fast aggregation [50]. In the case of a combined electrostatic and steric stabilization, a.k.a. electrosteric stabilization, a minimum zeta potential of ±20 mV is desirable [51]. However, drug nanosuspensions with zeta potentials below 20 mV (absolute) were physically stable in some earlier work [101,117,118], which could be explained by the adsorption of nonionic polymer or nonionic surfactant and ensuing steric effect alone. Hence, the use of zeta potential alone, especially for predicting the stability of drug nanosuspensions stabilized with combinations of polymers–surfactants, should be considered with caution [119,120,121,122].

The selection of an optimal stabilizer formulation is a laborious experimental task, yet an important one to produce a stable drug nanosuspension. A poorly formulated drug nanosuspension may undergo aggregation, Ostwald ripening, fast sedimentation of particles, and cake formation during milling/storage, which will lead to various issues in downstream processing of the respective suspensions, and poor product performance from the final oral solid dosages such as slow drug release [23,33,65,123]. As a general principle, if used at insufficiently low concentrations, stabilizers such as polymers and surfactants in drug nanosuspensions may not prevent aggregation, while their excessive use, especially for surfactants, can promote Ostwald ripening [53,54,100] or raise the viscosity so much that downstream processing may be negatively affected, e.g., inability to spray a drug nanosuspension in spray-drying and fluidized bed coating.

The first systematic investigations of the stabilizing capability of adsorbed polymers were carried out by Lee et al. [124,125]. A connection between the hydrophobicity of the polymer and the ability to stabilize drug nanocrystals was indicated [124]. In addition, differences in the surface energy between the particle and the polymer were found to play a role in the stabilization process [125]. Choi et al. [126] concluded that not only the surface energy, but also the specific interaction between the stabilizer and the drug appears to play important role. George and Ghosh [127] investigated the correlation between drug–stabilizer properties and critical quality attributes (CQAs) of drug nanosuspension formulations. Their study suggested that log*P* and fusion enthalpy of the drugs had a direct impact on the feasibility of a stable nanosuspension, and that the most likely candidate for WMM was a drug with high enthalpy and hydrophobicity. In contrast, in a more comprehensive study, Eerdenbrugh et al. [119] used 13 stabilizers at three different concentrations in wet-milled suspensions of nine drug compounds, and concluded that no correlation between physicochemical drug properties (molecular weight, melting point, log*P*, solubility, and density) and stable nanosuspension formation exists.

Recent modeling and experimental investigations [63,66,85,99,100,128,129] have suggested that the combined use of non-ionic cellulosic polymers such as hydroxypropyl cellulose (HPC), hydroxypropyl methyl cellulose (HPMC), etc. and surfactants, especially anionic surfactants such as sodium dodecyl sulfate (SDS), dioctyl sodium sulfosuccinate (DOSS), etc., can have synergistic stabilization effects on drug nanosuspensions. Bilgili et al. [101] demonstrated HPC–SDS combinations for adequate stabilization of five BCS Class II drugs, attributing the synergistic stabilization to an electrosteric mechanism, similar to that described in previous studies [65,99]. When SDS was used below the critical micelle concentration (CMC) to stabilize a griseofulvin nanosuspension along with HPC, the significant synergistic stabilizing action of HPC–SDS was attributed to enhanced drug wettability (lower surface tension and higher wetting effectiveness factor) and ensuing higher deaggregation effectiveness afforded by the presence of SDS, in addition to the steric stabilization afforded by HPC [122].

## 3. Preparation, Characterization, and Formulation of Drug-Laden Nanocomposites

### 3.1. Drying Processes for the Production of Nanocomposites

**Figure 5:**
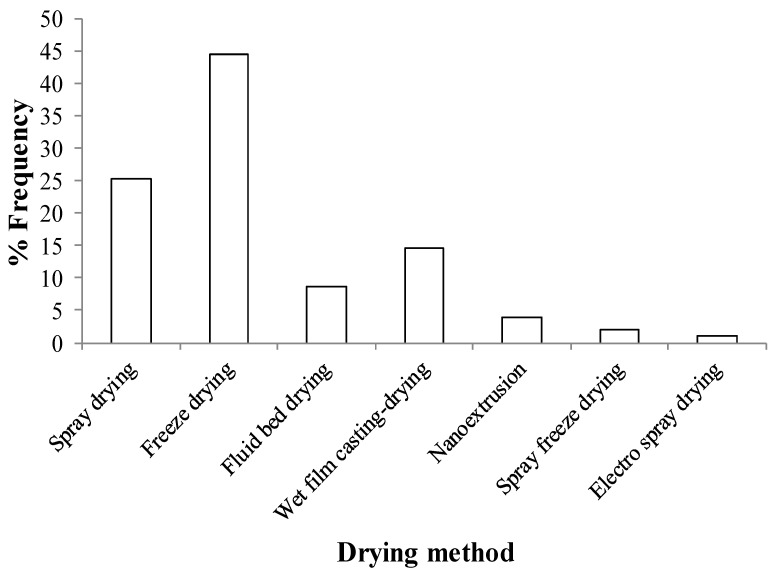
The usage frequency of various drying methods in the studies reported in Table 2. The sample size for the analysis here is 103, even though the number of publications in Table 2 is 92, because some studies compared two drying methods.

Drug nanosuspensions are dried to prepare drug-laden nanocomposites. Based on 92 studies published from 2012–2017, Table 2 lists the method of nanoparticle formation, drying method, and dispersant formulations used. It also presents the results from redispersion tests, including the characteristic particle sizes of the drug particles in the drug nanosuspension (before redispersion) and those sizes after redispersion of the nanocomposites in the redispersion medium. The data in Table 2 was analyzed and the usage frequency of various drying methods is presented in Figure 5.

Freeze drying is the most popular method for drying drug nanosuspensions into nanocomposites (Figure 5). During freeze drying, a.k.a. lyophilization, water is removed from a frozen sample by sublimation and desorption under vacuum. The freeze drying cycle can be divided into three steps: freezing (solidification), primary drying (ice sublimation) and secondary drying (desorption of unfrozen water). The general purpose of pharmaceutical freeze-drying is to achieve long-term stability of heat-labile active compounds in a formulation [195,196]. This process generates various stresses during the freezing and drying steps. So, protectants are usually added to the nanosuspension formulation to protect the nanoparticles from freezing and desiccation stresses, as well as to prevent aggregation during drying. Most commonly used dispersants include cryoprotectants such as trehalose, glucose, sucrose, and mannitol [197]. The immobilization of nanoparticles within a glassy matrix of cryoprotectant can prevent their aggregation and protect them against the mechanical stress of ice crystals [90,197]. Type–concentration of stabilizers (polymer–surfactant) and cryoprotectants, drug loading, and the drug:cryoprotectant ratio affect drug nanoparticle aggregation and redispersibility [74,197]. Usually, a freeze-thawing study should be realized before freeze drying to select the cryoprotectant which is best able to conserve the properties of the nanoparticles. The critical process conditions during freeze drying are the velocity of freezing with or without annealing, the pressure and shelf temperature, and the duration of each stage of the process i.e., freezing time, primary drying time, and secondary drying time [196,197].

Spray drying is the second most popular drying technique for converting nanosuspensions into dry powders (Figure 5). In spray drying, solutions or suspensions are atomized through a bi-fluid or pressure nozzle and sprayed into a drying chamber, and hot air passing co-currently dries the atomized droplets [198]. The particles formed upon evaporation of the liquid are further dried in the cyclone and separated into a collecting chamber. The critical process parameters during spray drying are droplet size, process air temperature, atomization air flow rate, suspension/solution flow rate, viscosity, and solids loading in the feed [89,199]. Vehring [89] provides an extensive review of pharmaceutical spray drying processes in general. Several investigations have demonstrated the formation of high drug-loaded, fast-dissolving drug-laden nanocomposite microparticles upon drying of drug nanosuspensions [27,85,121,185]. Spray-dried powders usually have a low bulk density and particle size, i.e. in the range of ~5­–50 µm, depending on the formulation [89,200].

**Figure 6:**
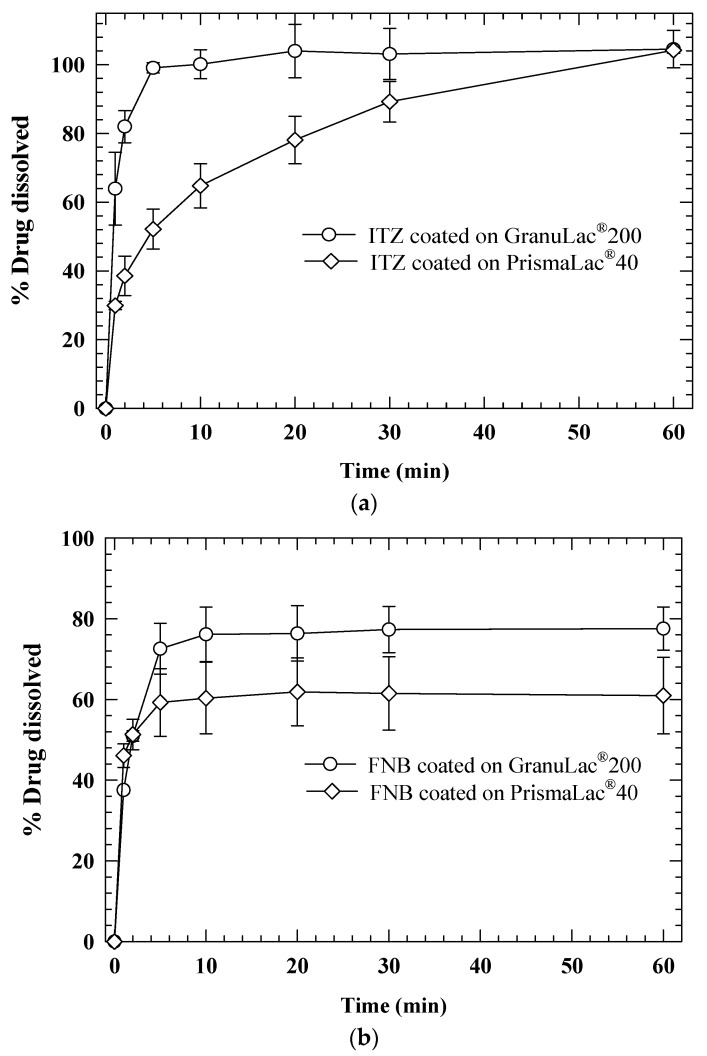
Comparison of drug release from the nanocomposite particles: (**a**) ITZ coated on PrismaLac 40 vs. GranuLac 200, (**b**) FNB coated on PrismaLac 40 vs. GranuLac 200, during the USP II dissolution test. Dissolution was performed using 7.2 mg/mL SDS solution for (**a**) and 2.88 mg/mL SDS solution for (**b**). (Figure adapted from Azad et al. [91] with permission from Elsevier, www.elsevier.com).

In fluid bed coating/drying, drug nanosuspension is atomized via a bi-fluid nozzle and sprayed onto fluidized carrier/substrate particles, also known as beads, and coating–drying of the nanoparticles on the beads occurs (bead layering) [64]. Usually, lactose or microcrystalline cellulose particles with sizes from 50–1000 µm are used as carriers. The suspension flow rate, atomization air pressure, fluidization air flow rate, and air temperature are controlled to prevent agglomeration of the coated particles. In an alternative processing route, i.e., fluid bed granulation/drying, drug nanosuspensions containing polymers, which serve as granulation binder fluid, can be sprayed onto a fluidized bed of excipients to form granules or agglomerates [130,192]. There are 7 papers on fluidized bed coating vs. two on fluid bed granulation (see Table 2). Hence, we focus on fluid bed coating here. Bhakay et al. [66,84] and Azad et al. [91,201] fluid bed coated nanosuspensions of griseofulvin and fenofibrate onto various grades of lactose carrier particles and investigated the impact of formulation and carrier size on the formation of redispersible nanocomposite particles. Knieke et al. [152] successfully coated sub-50 µm lactose carrier particles with fenofibrate nanosuspension without appreciable agglomeration, and prepared free-flowing powders for proper downstream processing. In spite of having a median particle size less than 100 µm, the final composite powders were free flowing, had a high bulk density, and exhibited fast fenofibrate release during the dissolution. Azad et al. [91] examined the impact of carrier particle size on redispersion and dissolution of itraconazole (ITZ) and fenofibrate (FNB) nanocomposites. The drug nanosuspensions were prepared by WMM with HPMC–SDS as stabilizers, followed by their coating onto the GranuLac 200 (*d*_50_: 27.7 µm) and PrismaLac 40 (*d*_50_: 321.1 µm) particles. As seen in Figure 6, the nanocomposite particles with GranuLac 200 exhibited significantly faster drug dissolution than those with PrismaLac 40. Finer carrier particles, owing to their higher surface area per unit mass and corresponding thinner coating layer (drug–polymer shell), provide faster drug release than coarser carrier particles.

Recently, a nanoextrusion process has been developed to disperse drug nanoparticles in a polymeric matrix (nanocomposites) in the form of extrudates using a variant of the traditional hot melt extrusion (HME) process [75,76,77,78]. Unlike a traditional HME process, the nanoextrusion process uses a wet-milled suspension of the drug as feed, along with an extrusion polymer, and disperses drug nanoparticles in the polymeric matrix while evaporating the water, thus yielding dry extrudates in the form of nanocomposites. Simply put, nanoextrusion acts as a continuous drier unlike the traditional HME process. This technique was first presented by Khinast et al. [75] as a one-step process for converting a stabilized nanosuspension into nanocomposite, where crystalline nano-titanium oxide was used as a model substance. In a follow-up study by Baumgartner et al. [76], crystalline phenytoin nanoparticles were used to demonstrate applicability to pharmaceutical products. Ye et al. [77] combined the use of high-pressure homogenization and extrusion for the production of nanocomposite with low drug concentration, i.e., 1–2%. Li et al. [78] prepared extrudates with nanocrystalline griseofulvin (GF) particles dispersed in the HPC matrix as a secondary phase (nanocomposites) and extrudates with amorphous GF molecularly dispersed within the Soluplus^®^ matrix (amorphous solid dispersion), demonstrating the versatility of the nanoextrusion process. Li et al. [87] milled the GF–HPC extrudates into various sieve cuts and examined the impact of matrix (particle) size on drug release; they observed that finer extrudate particles led to faster drug release, but such an impact quickly attains a plateau below ~200 μm. 

Besides the interest in the aforementioned drying methods that yielded powders/cakes or extrudates, there has also been an interest in incorporating drug nanoparticles into polymeric strip films (Figure 5). Thin strip films offer significant advantages for oral delivery of drugs, such as rapid disintegration and dissolution in the oral cavity, especially for geriatric–pediatric populations [202]. The wet film casting–drying commonly used with soluble drugs has been applied to dry drug nanosuspensions in polymeric strip films, which encapsulate drug nanoparticles in a polymeric matrix uniformly (nanocomposite) [79,203]. In this method, drug nanosuspensions are mixed with aqueous solutions of HPMC E15LV, HPMC K4M, or pullulan, along with a plasticizer for desired viscosity; the resulting slurry is cast into film using a casting knife to control the film thickness, followed by drying. The resulting film is cut into different sizes according to the required dose. Drug nanoparticles prepared by both top-down and bottom-up approaches have been incorporated into thin polymeric strip-films that exhibit significantly enhanced drug release and acceptable content uniformity, even for low drug doses [40,79,109]. Sievens et al. [79] wet-cast and dried a slurry formed by mixing griseofulvin, fenofibrate, and naproxen nanosuspensions prepared by WMM with aqueous HPMC–glycerin solutions. The films exhibited good redispersibility for all drugs. The dissolution testing was only performed for fenofibrate; films exhibited faster drug release compared with the as-received drug, physical mixture, and the compact of the drug. Susarla et al. [131] investigated the impact of convection drying parameters such as air velocity and temperature, as well as film precursor viscosity on film properties and redispersion of griseofulvin nanoparticles. The griseofulvin nanoparticles were fully recovered upon redispersion in water from all convection-dried films, suggesting that the film formation process, including faster drying, did not lead to irreversible drug nanoparticle aggregation. Krull et al. extensively studied the impact of different plasticizers [81], polymer molecular weight [82], and the impact of drug loading [83] in a series of papers on redispersion of BCS class II drugs from polymeric strip films and drug release during in vitro dissolution. In a separate study, Krull et al. [162] prepared griseofulvin nanoparticle laden pullulan–xanthan gum films. Thinner films, films with lower xanthan gum loading, and smaller drug nanoparticles led to faster drug release from the films, while drug loading had no discernible effect within the range studied.

### 3.2. Characterization of the Nanocomposites

Nanosuspension characterization methods have been discussed in several review papers [23,28,94]. In this section, we briefly review various characterization methods for the characterization of the nanocomposites. Several methods are routinely used, except redispersion methods and drug wettability testing, which will be discussed in more detail. 

#### 3.2.1. Particle Sizing

Particle size of nanocomposite particles is usually measured by laser diffraction using e.g., Sympatec Rodos/Helos particle sizer, Beckman Coulter, or Malvern Mastersizer, in the dry particle sizing mode. Approximately 1 g of nanocomposite powder was placed on the chute of a Rodos dispersing system and particle size was measured at an optimum dispersing pressure to avoid attrition of nanocomposite particles [27,86]. The particle size of nanocomposite particles after redispersion in a liquid medium and drug particle size in nanosuspensions can be measured using the liquid module of Beckman Coulter or Malvern Mastersizer by laser diffraction (e.g., [8,66,85,152]). Malvern zetasizer and Delsa nano are also used to measure size of nanosuspensions and redispersed nanocomposite particles via dynamic light scattering (DLS) (see e.g., [103,150,151,182]). Since DLS can measure particle sizes accurately up to few microns, it cannot measure coarse aggregates or clusters present in the nanosuspensions or redispersed nanocomposite particles. On the other hand, laser diffraction instruments cannot accurately determine particle sizes below 100 nm. Unless median drug particle size is smaller than 100 nm, laser diffraction is the first method of choice, as it can detect the primary particles as well as large aggregates/clusters in a given sample. Otherwise, DLS alone or in combination with an orthogonal method like scanning/transmission electron microscopy coupled with image analysis could give valuable information (e.g., [103]).

Atomization of nanosuspensions via a bi-fluid or pressure nozzle is an integral part of spray drying and fluid bed coating/drying processes. Malvern Spraytec/Insitec RTSizer or similar laser-diffraction based system can be used to measure size of droplets emanating from a nozzle (e.g., [204,205]), which allows formulators to examine the impact of atomization air pressure, nanosuspension flow rate, and viscosity on droplet size. Once the droplet size is known at a small scale, it should be maintained across different scales to ensure proper scale-up of the drying process.

#### 3.2.2. Scanning Electron Microscope (SEM) Imaging

The morphology and structure of nanocomposites can be examined under a scanning electron microscope (SEM). A few nanocomposite particles can be placed on a carbon tape which is stuck onto an SEM stub, and images are taken at different magnifications under SEM (e.g., [27,206]). The cross-section of nanocomposite particles can be seen by embedding the nanocomposite particles in epoxy, slicing the epoxy using a microtome, and placing a thin cut of epoxy under SEM (e.g., [207]). Another method for the visualization of cross-section of nanocomposite particles is to spread the particles onto a glass slide, cut them with a sharp knife, and focus on the broken particles under SEM (e.g., [66,207]). 

Elemental analysis of nanocomposite powders can be done by energy dispersive X-ray analyzer to confirm uniform dispersion of API in the nanocomposite particles. An electron microscope with a detector for characteristic X-rays, i.e., energy-dispersive X-ray spectroscopy (EDS), is a powerful analytical method for quantitative and qualitative surface analysis. Generated X-rays inside the SEM are characteristic for each atom in the periodic table (excluding He and H), and their detection by EDS allows for measuring the elemental composition of the sample. The amount of emitted X-rays from each element is directly proportional to its concentration (mass or atomic fraction) in the sample. EDS analysis will only work if an element present in the drug molecule gives a distinct X-ray signal [207]. For example, Azad et al. [27] employed EDS to show the presence of milled superdisintegrant particles along with fenofibrate nanoparticles in nanocomposite particles.

The suspension obtained after redispersion of nanocomposites particles can be observed under SEM by placing a drop of the suspension on a silicon chip [8,86]. The silicon chip is placed onto a carbon tape stuck onto a SEM stub, and dried in a vacuum oven before imaging using SEM. The objective of SEM imaging after redispersion of nanocomposite particles in water is to look for nanoparticles and nanoparticle aggregates [66,85].

#### 3.2.3. Drug Crystallinity

The crystallinity of the drug in nanocomposite particles is usually examined via X-Ray diffraction (XRD) (e.g., [183,208]) and differential scanning calorimetry (DSC) (e.g., [133,209]). The crystallinity of the drug in nanocomposites is affected by the nanosuspension preparation and drying method, as well as the formulation, especially drug–polymeric dispersant interactions. For most wet media milled drugs dried into nanocomposite particles with polymeric dispersants, the diffractograms show that the peak positions are preserved with some broadening and intensity reduction due to the formation of fine nanocrystals, defect formation, and even some partial amorphization [210,211,212]. For similar reasons, DSC thermograms clearly show a distinct melting endotherm, albeit with a melting point depression and reduced fusion enthalpy, as compared with as-received drug and physical mixtures [22,27,213]. Freeze-drying of drug nanosuspensions prepared by top-down approaches could either preserve drug crystallinity or result in some reduction in crystallinity, depending on the concentration/type of dispersant used [179,182,190]. Freeze drying of drug nanosuspensions prepared via LASP tends to result in a pronounced loss of crystallinity, and even complete amorphization [194,214,215].

#### 3.2.4. Redispersion Methods

Drug nanoparticles in the dispersant matrix of nanocomposites must be released into the dissolution medium in vitro or in vivo, preferably in the form of discrete primary nanoparticles, so that large surface area of the nanoparticles could allow for fast, immediate drug release. Hence, developing an understanding of the redispersion phenomenon and nanoparticle release mechanisms could significantly help robust nanocomposite formulation development [66,84,92]. Redispersion testing entails dispersing nanocomposite particles in water or a physiologically relevant fluid upon application of agitation/shear, so that drug nanoparticles are recovered in the fluid [66,216]. Table 2 shows that common agitation methods include sonication, magnetic stirring, pipette stirring, paddle stirring, manual shaking, as well as in-situ shearing/agitation in the sample cell of laser diffraction equipment. Unlike dissolution testing, redispersion testing judiciously uses a dispersion fluid volume–nanocomposite mass so that all dispersants present in the nanocomposite dissolves, while drug nanoparticles simply disperse in the fluid without significant extent of dissolution; hence, their particle sizes can be measured.

In redispersion studies, it is customary to compare the particle size of the milled suspension with that of the suspension obtained from the redispersion of the nanocomposites in water. This comparison has been used to assess the recovery and redispersion behavior of drug nanoparticles [66,67,92,147,217]. If the redispersed suspension contains water-insoluble dispersant particles, these particles can be removed by centrifugation or filtration to avoid interference during particle size measurements. If possible, either filtration should be avoided, because drug particle aggregates and agglomerates may also be removed in this process, or filter opening size should be carefully chosen to separate only water-insoluble dispersants. The supernatant obtained after centrifugation can be used to measure the particle size of the redispersed suspension. The centrifugation time and speed should be optimized such that drug aggregates are present in the supernatant. Another approach is to analyze the supernatant by dissolving the drug in a solvent, measuring the concentration via UV or HPLC, and calculating the fractional recovery by comparing it with the original amount of drug concentration present in the nanocomposite particles [92].

A cursory look at the literature prior to 2012 reveals that many studies on the drying of pharmaceutical nanosuspensions did not consider the redispersion and recovery of nanoparticles at all [34,59,60,64,69,70,218,219,220]. Redispersion testing was performed, but little to no detail was provided in other studies [33,65,197,221,222]. Yet another group of studies investigated the recovery of polymeric nanoparticles, silica, and nanocapsules without drugs [58,61,217,223]. In a smaller group of studies, the recovery of drug nanoparticles during redispersion was investigated for spray-dried [62,63], spray-freeze dried [67], freeze-dried [74], and fluid-bed dried [63] nanocomposites. These studies described a single redispersion method without investigating the potential impact of redispersion method itself, due to differences in agitation/shearing conditions. Table 2 shows that 73% of the nanocomposite formulations in 2012–2017 studies were subjected to redispersion testing. The data in a 2014 review paper by Chin et al. [55] suggest this ratio to be 40% (33% for studies before 2011). This comparison shows that there is significantly more interest in developing an understanding of the redispersion behavior of the nanocomposites in recent studies.

To the best of authors’ knowledge, there are only two studies [66,92] which solely investigated the redispersion of drug-laden nanocomposites using multiple redispersion test methods. Bhakay et al. [66] studied the impact of different redispersion methods on particle size of drug-laden, core (lactose)–shell nanocomposite microparticles. About a gram of the nanocomposite particles were weighed and dispersed in 30 mL water for 2 min. The maximum amount of the drug that can dissolve in water during the redispersion test in their method was very small (e.g., about 0.2% of drug particles). Dispersants such as HPC, SDS, lactose, and mannitol in nanocomposite particles dissolve in water and the particle sizes obtained from particle size instruments were mainly the sizes of undissolved drug particles and their clusters. Fast redispersion and recovery of nanoparticles (e.g., within 2 min) under low agitation conditions could be a desirable attribute of robust BCS Class II drug formulations in immediate release, solid dosage forms. Gentle pipette stirring (manual), magnetic stirring at 100 rpm, stirring with an overhead laboratory stirrer at 200 and 1200 rpm, and sonication in an ultrasonic bath were used. After dispersing the nanocomposite particles in water or buffer solutions, an aliquot of the sample was taken while the sample was being agitated, and the particle size was measured by laser diffraction. This study established that the nanosuspension formulation (dispersant type/concentration) has more impact on the redispersed particle size than the agitation/shearing method. In principle, any of the above redispersion methods may be used to rank different dispersant formulations according to their capabilities of releasing nanoparticles. Different redispersion methods had only a slight impact on the recovery of nanoparticles when SDS was present either in the formulation or in the redispersion medium (water). Sonication appears to be more effective than stirring-based methods in deaggregating the clusters and aggregates formed during the redispersion in water.

**Figure 7:**
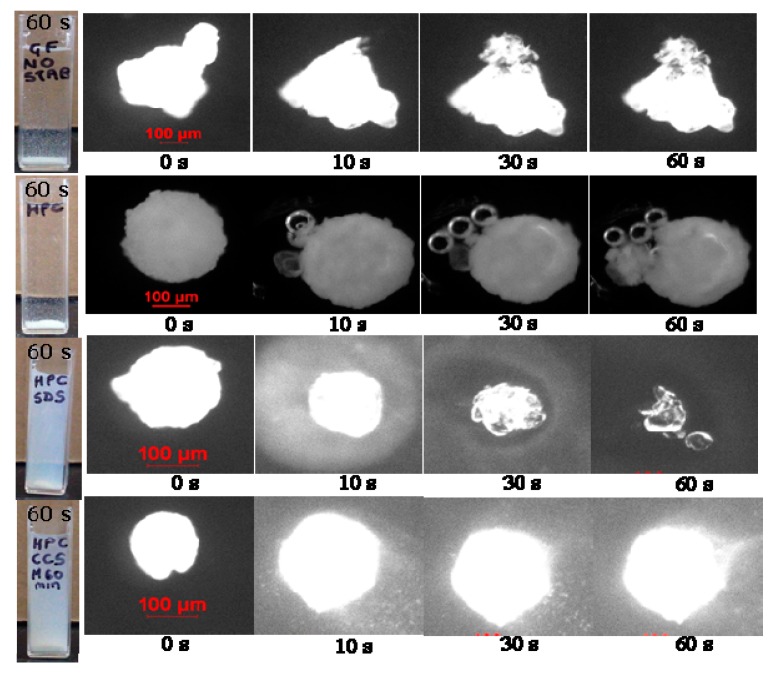
Images of redispersion dynamics of griseofulvin (GF) nanocomposite particles, which were prepared by fluidized bed coating of GF nanosuspension on Pharmatose carrier particles with various dispersants, in quiescent water (no external agitation/shear) (**left panel**), and after addition of a drop of water on a single nanocomposite particle, visualized under optical microscope (**right panel**). Nanocomposite formulations in the figure from top to bottom contain GF nanoparticles without stabilizers, GF nanoparticles with HPC, GF nanoparticles with HPC–SDS, and GF with HPC–croscarmellose sodium (CCS) milled for 60 min. (Adapted from Bhakay et al. [92] with permission from Springer Nature, www.springernature.com).

Bhakay et al. [92] have assessed the use of quiescent redispersion methods in comparison to agitated redispersion methods. Approximately 100 mg nanocomposite particles were dispersed in 4 mL water in a cuvette, as shown in Figure 7 (left panel), with the dimensions of 1.2 × 1.2 × 4.6 cm (L × W × H). As the nanocomposite particles descend and sediment, they release drug particles that can be observed visually and quantified by determination of turbidity, particle size, and drug assay. Several pictures were captured during the descent of nanocomposite particles every few seconds. During the settling, nanocomposite particles with no stabilizers, or with HPC alone, did not release significant amount of nanoparticles, as signified by low turbidity and low drug nanoparticle concentration in the supernatant. In a separate experiment, nanocomposite particles were dispersed in water kept in a cuvette without external agitation, and particle size was measured via dynamic light scattering (DLS) with Delsa nano. In a separate method, nanocomposite particles were redispersed in 4 mL water for 5 min and the resulting suspensions were centrifuged for 5 min to remove particles that did not redisperse. Centrifugation removed the large particles and clusters and mitigated high scattering during dynamic light scattering size measurements which would otherwise cause imprecise results. Another method is to study redispersion dynamics via DLS by dispersing the nanocomposite particles in water and measuring the size to develop an understanding of how larger particles redisperse over a period of time. Redispersion phenomena can also be visualized under optical microscope by adding a drop of water or buffer solution to a nanocomposite particle and capturing images at different time intervals, as shown in Figure 7 (right panel). The appearance of a “cloud” around the nanocomposite particle was a signature of drug nanoparticle recovery, and those formulations that exhibit such clouds also exhibited high turbidity generation during the sedimentation (see Figure 7, left panel). Overall, Bhakay et al. [92] concluded that both quiescent redispersion and agitated redispersion methods yielded similar rank-ordering of the dispersants used in the nanocomposites; hence, both types of tests could be used for screening/optimizing dispersants for fast-dissolving drug nanocomposite formulations.

#### 3.2.5. Dissolution Testing

Nanocomposite samples having drug amounts equivalent to saturation solubility, or lower than saturation solubility of BCS Class II drugs in the dissolution medium have been used preferentially because such non-sink conditions have been shown to better discriminate between various nanocomposite formulations [59,169,224,225]. Water, aqueous surfactant solutions, buffer solutions, or other simulated biological fluids can be chosen as dissolution media. USP Type I apparatus (basket method) [150,159], USP Type II apparatus with a rotating paddle [66,135,146], and USP IV flow-through cell apparatus [79,131,153] were used to measure drug release from nanocomposites. Aliquots can be taken manually or in an automated fashion, at different time intervals to analyze percent drug dissolved. The amount of drug dissolved can be determined by measuring absorbance using UV spectroscopy or HPLC.

#### 3.2.6. Drug Wettability

Wettability of drug particles and wettability enhancement upon use of various dispersants [78,122,226] can be examined via liquid penetration into a drug powder bed, also known as the *modified Washburn method* [227,228]. A typical powder tensiometer, e.g., Attension Sigma 700 set-up (Biolin Scientific, Linthicum, MD, USA), consists of a sample holder in the form of a cylindrical metallic tube with small holes at the bottom, as well as a hook at the top of the cover equipped with screw threads. About ~1 g of drug powder was packed uniformly into the tube before each measurement (e.g., [78]). A filter paper was placed at the perforated end of the sample holder to support the drug powder sample. A petri dish containing liquid, typically water or a dispersant solution, was placed below the perforated end of the holder on the mechanical platform. The powder tensiometer measures the mass of liquid penetrated into the drug powder bed as a function of time. The apparent shear viscosity and surface tension of the liquids must be known or measured. The contact angle for the deionized water/dispersant solution and drug can be determined using the modified Washburn equation, which provides a relationship between liquid penetration rate and contact angle *θ*as follows:

(1) $$T=\left(\frac{\eta }{C\rho^{2}\gamma \cos\theta }\right)M^{2}$$

where *T*, *M, η*, *ρ*, and *γ* are time after contact, mass of the liquid penetrated into the drug powder bed, viscosity of the liquid, density of the liquid, and surface tension of the liquid, respectively. *C* is a characteristic parameter of the drug powder sample. It could be determined independently using a completely wetting liquid such as hexane, heptane, etc. In examining the impact of various dispersants on wettability, the same drug powder is used as the powder sample; hence, *C* remains relatively invariant for different dispersant solutions and deionized water under similar packing conditions. One can therefore calculate the ratio of cos*θ*_d_/cos*θ*_w_, i.e., wetting effectiveness factor, as a crude measure of wettability enhancement by the dispersants. Here, *θ*_d_ is the contact angle between drug and the dispersant solution, and *θ*_w_ is the contact angle between drug and deionized water. The wettability enhancement with different dispersants on the wetting of drug particles may be assessed by comparing this ratio, taking the wettability by water as a basis of comparison [78]. The slope of the modified Washburn equation is obtained by fitting the linear region of a liquid penetration curve. Using the slope for different stabilizer solutions and water, cos*θ*_d_/cos*θ*_w_ is calculated. Dispersants, especially surface-active adsorbing polymers and surfactants, improve drug wettability, and thus, help deaggregation during WMM [122] and enable faster redispersion and drug release from drug nanocomposites [78,87].

### 3.3. Nanocomposite Formulations and Functionalities of Dispersants

**Figure 8:**
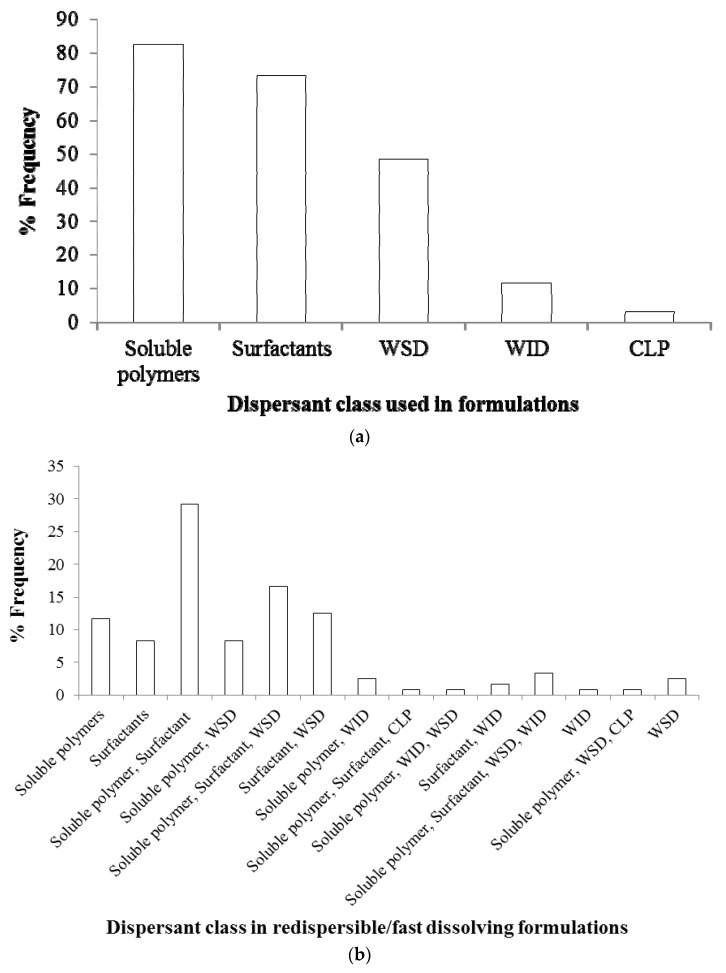
The usage frequency of (**a**) various classes of dispersants and (**b**) classes of dispersants in the formulations that led to formation of redispersible nanocomposites in the studies reported in Table 2. The sample size for the analysis here is 120, even though the number of publications in Table 2 is 92, because some studies investigated more than one drug. WSD: other water-soluble dispersant (besides soluble polymer–surfactant), WID: water-insoluble dispersant, CLP: crosslinked polymers.

Table 3 classifies various dispersants and their functionalities–action mechanisms in drug nanosuspensions–nanocomposites, while Figure 8 presents their usage frequency in nanocomposite formulations. In the statistical analysis of Table 2 data with various dispersants used in a given study/formulation, a redispersible formulation was regarded as either the one that led to the finest drug particle size upon redispersion of the nanocomposites (see Table 2), or that led to the fastest dissolution if redispersion testing was not performed in that particular study. In the latter case, similar to Eerdenbrugh et al. [34,59], redispersibility was not directly studied, but inferred from the dissolution testing. Different classes of dispersants, dispersant loading, drug type, drug loading, ratio of drug:dispersant concentration, drying method, and particle formation methods have an impact on the redispersion of nanoparticles from nanocomposites and drug release. Some of the salient features and impacts of various classes of dispersants are presented below.

#### 3.3.1. Impact of Soluble Polymers on Redispersion–Drug Release

Figure 8a shows that about ~83% of the nanocomposite formulations used soluble polymers either alone or in combination with other dispersants. Soluble polymers include cellulosic polymers, polyvinyl pyrrolidone, polyethylene glycol, etc. [33,86]; different grades of HPC, HPMC and PVP are the most popular (refer to Table 2). The typical use levels of these polymers are 1–7.5% *w*/*v* in nanosuspension formulations. They usually play a dual rule: firstly, being soluble dispersants that adsorb on drug nanoparticles, they act as steric stabilizers during the preparation of drug nanosuspensions [23,31,100]. Secondly, they serve as primary matrix/film former in the nanocomposites encapsulating/covering drug nanoparticles upon drying of drug nanosuspensions and mitigate nanoparticle aggregation during the drying [28,55]. Moreover, higher viscosity of the drug nanosuspension upon use of soluble polymers could help to retard aggregation due to slower Brownian motion during storage, and even mitigate phase separation-induced aggregation during drying [100], depending on the specific drug–polymer and their mass ratio.

Lee [33] prepared 16 wt.% drug (unspecified) nanosuspensions by WMM with HPC as the stabilizer, and spray-dried nanosuspensions to obtain nanocomposite particles. Increasing HPC concentration from 0.38–3.08 wt.% in suspension resulted in greater HPC adsorption onto the drug surface, and produced nanosuspensions with smaller particle size during milling. At a high concentration of HPC (3.08%), polymer adsorption on drug surface seemed to be sufficient to cover the drug surface and prevent aggregation of nanoparticles by steric stabilization during milling. It took about 25 h to completely redisperse the nanocomposite particles back into drug nanoparticles in water. Lee [33] attributed this slow nanoparticle recovery solely to aggregation during drying, perhaps due to the low (~1:5) mass ratio of HPC:drug. Other possible explanations include the formation of drug nanoparticle aggregates during storage prior to spray-drying (no stability study provided in Lee [33]), aggregation due to increased capillary forces during drying [34,88], and slow wetting and penetration of water into the spray-dried particles, besides the relatively slow dissolution of HPC. While Lee [33] did not study drug release, Li et al. [86] coated wet media milled griseofulvin (GF) suspensions stabilized with HPC onto Pharmatose carrier particles via fluidized bed coating/drying, and examined the impact of HPC concentration on GF release. The findings were somewhat similar to those of Lee [33], except that most of the drug aggregates already formed during the milling–storage, which led to poor redispersion and slow dissolution; immediate drug release was not achieved. Li et al. [86] demonstrated that higher HPC concentration led to smaller aggregates during milling and after redispersion, which in turn enabled faster drug release from the nanocomposites; however, since aggregation could not be suppressed completely, immediate drug release was not achieved. 

In other studies such as He et al. [160], Ho and Lee [120], and Kumar et al. [139], drug nanoparticles were recovered from nanocomposites with soluble polymers that serve as stabilizers/dispersants. He et al. [160] formed nanoparticles of teniposide in the presence of PVP K30 by antisolvent precipitation followed by freeze drying; Ho and Lee [120] milled naproxen down to the nanosize domain with HPC and formed nanocomposite particles by electrospray drying; and Kumar et al. [139] wet-milled naproxen particles with HPMC E15 followed by spray drying to form nanocomposite particles. While the above-mentioned studies used different drugs and different particle formation–drying methods, the nanocomposites completely redispersed into nanoparticles during manual redispersion or sonication in less than 5 min. Ho and Lee [120] did not study dissolution of spray-dried nanocomposites. He et al. [160] and Kumar et al. [139] corroborated the redispersion results with dissolution; teniposide and naproxen nanocomposite particles formulated with PVP K30 and HPMC E15 showed significantly improved dissolution.

The aforementioned studies demonstrate that (i) preventing aggregation in drug nanosuspensions is a necessary condition for fast redispersion and drug release from the nanocomposites; (ii) soluble polymers may not be able to stabilize drug nanoparticles, regardless of the concentration used, leading to the formation of aggregates in the nanosuspensions; (iii) depending on the drug:polymer ratio, aggregates may also form during drying, which will cause inferior dissolution enhancement; and (iv) higher polymer concentration may alleviate the aforementioned issues at the expense of reduced drug loading in the nanocomposites. The upshot of these results is that other classes of dispersants, in addition to soluble polymers, may help to resolve the aforementioned issues; hence, it is no surprise to see only ~12% of the redispersible formulations used soluble polymers alone (Figure 8b).

#### 3.3.2. Impact of Surfactants on Redispersion–Drug Release

~73% of the formulations in Table 2 used surfactants alone or in combination with other dispersants (Figure 8a). Being surface-active agents, surfactants reduce the surface tension of suspension liquids, help to disperse drug particles in suspension liquid by enhancing drug wettability, and help to stabilize drug nanosuspensions either alone or in combination with polymers [23,28,55,163]. They also impart wettability enhancement to the nanocomposites which help to speed up redispersion and drug nanoparticle recovery [78,86]. Charged surfactants such as SDS and DOSS stabilize drug nanosuspensions by electrostatic stabilization, while polymeric surfactants like poloxamers, TPGS, tween 80 etc. stabilize nanoparticles by steric repulsion. The typical use levels of surfactants are between 0.05–5% *w*/*v* in drug nanosuspensions. Surfactant usage below critical micellar concentration (CMC) is preferred, because at concentrations greater than CMC, drugs tend to dissolve in the micelles, which may lead to particle size growth via Ostwald ripening during storage of the nanosuspensions [53,54].

Eerdenbrugh et al. [34] wet media milled 9 model BCS Class II drugs with Vitamin E TPGS at 25 wt.% (with respect to drug) and dried the suspensions into nanocomposite particles by freeze drying and spray drying. Drugs with more hydrophobic surfaces formed aggregates during drying, and compromised the dissolution rate as compared to corresponding milled nanosuspensions. Other drugs formed redispersible nanocomposite particles with a polymeric surfactant alone. Bhakay et al. [66] reported poor redispersion from one-month aged nanocomposites prepared by fluid bed drying/coating of GF–SDS nanosuspension, while redispersibility was achieved by GF–HPC–SDS nanocomposites. Li et al. [86], for the same type of nanocomposites, found that when 0.5 wt.% SDS w.r.t. GF was used, significant aggregation occurred during the drying, which led to very slow GF release. The above findings, along with the observation that only ~8% of the redispersible formulations in Table 2 have surfactants alone (Figure 8b), suggest that other classes of dispersants, in addition to surfactants, must be considered to develop a more general approach for development of redispersible, fast-dissolving nanocomposite formulations.

#### 3.3.3. Impact of Soluble Polymer–Surfactant on Redispersion–Drug Release

Figure 8b shows that ~29% of the redispersible formulations had soluble polymer–surfactant, which is the most widely used strategy for formulating drug-laden nanocomposites based on the data in Table 2. Combinations of HPC–SDS, HPMC–SDS and PVP–SDS are some of the most common choices for forming redispersible nanocomposite particles. Besides the beneficial effects and mechanisms of action for the soluble polymers and surfactants mentioned in Section 3.3.1 and Section 3.3.2, their combined use could lead to synergistic effects in drug nanosuspension stabilization, mitigation of nanoparticle aggregation, and fast redispersion–drug release from the nanocomposites. For example, when anionic surfactants along with soluble polymers were used, electrosteric stabilization of the drug nanosuspensions, as well as faster deaggregation during the milling due to enhanced wettability of the hydrophobic drug particles, occurred (refer to Section 2.2.). Bhakay et al. [66], Niwa et al. [7], and Basa et al. [65] reported the positive impact of polymer–surfactant combination in the formation of redispersible nanocomposite particles for different drugs and drying methods. In general, the stabilization of drug nanosuspensions and fast redispersion/drug release require much less soluble polymer–surfactant compared to the case when either soluble polymers or surfactants are used alone. The use of less dispersants in the nanocomposite has several benefits, such as reduced extent of Ostwald ripening (at reduced surfactant loading) and higher drug loading in the nanocomposite.

**Figure 9:**
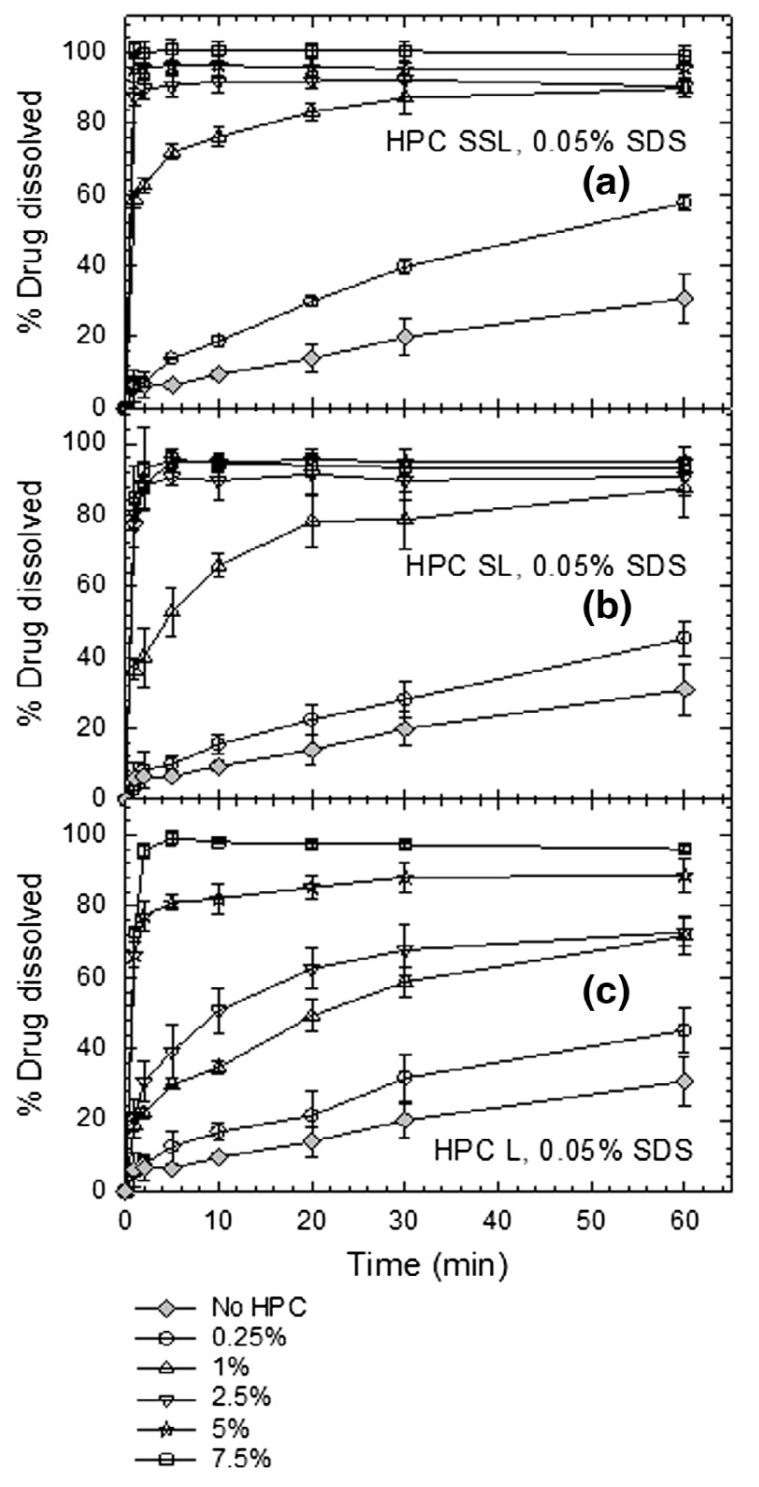
Drug (GF) release profiles of the nanocomposites prepared using wet-milled GF suspensions containing 0.05% SDS and HPC with different molecular weights of HPC: (**a**) HPC SSL (40 kDa); (**b**) HPC SL (100 kDa); and (**c**) HPC L (140 kDa) (Adapted from Li et al. [86] with permission from Elsevier, www.elsevier.com).

A systematic and comprehensive study was carried out by Li et al. [86] to examine the impact of HPC molecular weight and loading, SDS concentration, and combination of HPC–SDS on redispersion and dissolution performance of fluidized bed coated Pharmatose with wet-milled griseofulvin (GF) suspensions. In the absence of SDS, even at 7.5% HPC (any grade), GF nanoparticles severely aggregated in the wet-milled suspension, and dissolution from the nanocomposites did not achieve fast, immediate release, signifying the criticality of the surfactant. Figure 9 shows the impact of HPC molecular weight, in combination with 0.05 wt.% SDS, on dissolution of griseofulvin from nanocomposite particles. Immediate drug release was achieved when low molecular weight HPC SSL was used ≥1% *w*/*w* concentration (w.r.t. water) along with 0.05% *w*/*w* SDS. The highest molecular weight grade of HPC (HPC L) entailed the use of high concentrations of polymer (7.5% *w*/*w*) to immediately release drug nanoparticles despite of addition of 0.05% SDS. Besides showing the positive impact of polymer–surfactant combination, their study exemplifies some general trends: (i) higher dispersant concentration enabled faster drug release, albeit at a reduced drug loading; (ii) use of SDS at a small concentration (0.05%) was necessary to form almost aggregate-free nanosuspensions and mitigate aggregate formation during drying; (iii) proper stabilization of drug suspensions is a necessary condition for fast drug nanoparticle recovery and fast drug dissolution from the nanocomposites, but not sufficient because aggregation may also occur during drying, and nanoparticle recovery from the matrix of nanocomposites is controlled by wettability and dissolution of the dispersants; and (iv) immediate drug release was achieved when a lower molecular weight (SSL grade) HPC ≥1% concentration was used along with 0.05% SDS. 

While polymer–surfactant combinations appear to be the most widely used formulation strategy (Figure 8b), additional dispersant classes may be needed for a variety of reasons. Surfactants may pose challenges such as particle growth via Ostwald ripening during milling and/or storage [53,54,100,229], and for anionic surfactants, incompatibilities with other ionic molecules, sensitivity to pH, salt or temperature changes, and GIT irritation [230,231]. Another potential issue with surfactants is that they can be toxic if used in excess [230], especially in inhalation and parenteral applications [232,233,234]. Surfactants can be chosen from the handbook of pharmaceutical excipients which provides the typical use levels for different administration routes [235]. Additionally, a list of inactive ingredients (excipients including the surfactants) used in approved drug products is available on the U.S. Food and Drug Administration (FDA) website [236]. This list provides information on dosage form, route of administration, and maximum potency of the excipient per unit dose in already approved products. For new surfactants or different usage (dosage form/route of administration) of existing surfactants, FDA and The International Council for Harmonisation (ICH, Geneva, Switzerland) guidance documents lay out toxicity studies to be performed in rodent species to define use levels (readers are referred to [237] and the ICH guidance documents cited therein).

Some of the aforementioned issues can be mitigated by reducing the surfactant loading using streamlined and material-sparing formulation approaches (e.g., [100,238,239]), and by the combined use of polymers–surfactants which allow for lower surfactant loading. In fact, Ostwald ripening issue can be practically resolved by simply adding/mixing most or all of the surfactant with the nanosuspension containing drug–polymer right before the drying step. Another formulation strategy is to minimize the use of surfactants or eliminate their use completely by making use of other classes of dispersants. In combination with soluble polymer–surfactants, the use of other dispersant classes can reduce the surfactant loading required otherwise, while still ensuring, or even improving the performance of the nanocomposites. Alternatively, surfactants can be completely replaced by additional dispersant classes. The use of water-soluble dispersants (WSD) [197], water-insoluble dispersants (WID) [59], and crosslinked polymers (swellable, water-insoluble dispersants) [8,27] will be covered below.

#### 3.3.4. Impact of Other Classes of Dispersants

Water-soluble dispersants (WSD) refer to sugars, sugar alcohols, cyclodextrin, and some proteins present in the nanocomposites other than soluble polymers and surfactants. They dissolve on contact with water and aid in breakage/erosion of the nanocomposite matrix, as well as drug particle aggregates during redispersion/dissolution [28,55,138,152]. WSD are the most widely dispersants after polymers and surfactants (Figure 8a), and usage frequency of polymer–surfactant–WSD is second to that of polymer–surfactant in redispersible formulations (Figure 8b) because, having low molecular weight, WSD dissolve faster than soluble polymers, thus creating pores in nanocomposite matrix and facilitating polymer dissolution and drug nanoparticle release [152]. During freeze drying, sugars and sugar alcohols act as cryoprotectants by reducing ice formation during freezing, and thereby prevent formation of nanoparticle aggregates [197,240]. As can be seen from Figure 8b, they are rarely used alone because they cannot stabilize drug nanosuspensions—unlike polymers/surfactants—and they are relatively poor film formers. WSD:drug mass ratio must be set relatively high (for some drugs up to 10:1 in freeze drying [240]) so that they can physically separate the drug nanoparticles and prevent their aggregation. They are almost exclusively used along with polymer/surfactants and polymer–surfactants. For example, even with 1:1 mannitol:griseofulvin (GF) mass ratio, GF–HPC–mannitol nanocomposites prepared by either fluid bed coating [8] or spray drying [27] could not achieve fast, immediate release in the absence of a surfactant although addition of Mannitol significantly improved the drug release rate. Hence, WSD concentration must be optimized, or alternative classes of dispersants besides polymers/surfactants should be considered.

Water-insoluble dispersants (WID) such as microcrystalline cellulose, anhydrous dicalcium phosphate, colloidal fumed silica, and clay/montmorillonite particles prevent direct contact of drug nanoparticles with each other, thus preventing the formation of aggregates during drying and aid in redispersion [59,192]. Eerdenbrugh et al. [59] used WID such as Avicel PH101 (microcrystalline cellulose), Fujicalin (anhydrous dicalcium phosphate), and Aerosil 200 (colloidal fumed silica), as well as a polymeric surfactant (Inutec SP1). Nanosuspensions of phenylbutazone, itraconazole, and cinnarizine were prepared in an aqueous solution of 25 wt.% Vitamin E TPGS (surfactant) and spray-dried after the addition of the aforementioned dispersants. Among all three WID examined, only Aerosil 200 improved dissolution performance of all spray-dried powders, which was attributed to its large surface area and good matrix forming capability. However, the best dissolution performance resulted from the use of Inutec, i.e., the polymeric surfactant. Unfortunately, there is no comparative assessment of Aerosil 200 as a WID with commonly-used WSDs such as sugars and sugar alcohols. Hence, the use of such insoluble dispersants warrants further investigation.

Crosslinked polymers (CLP), especially wet-milled superdisintegrants [8,27,241], form a novel class of dispersants which erode/disintegrate the nanocomposite matrix by swelling on contact with water and release nanoparticles during redispersion–dissolution. Azad et al. [27] and Bhakay et al. [8] have used common superdisintegrants such as SSG, CCS, and CP as a novel class of dispersants with the goal of replacing surfactants in nanocomposite formulations. Superdisintegrants were wet-milled along with griseofulvin particles in the presence of HPC; the resultant suspension was either fluid bed coated onto Pharmatose carrier particles [8] or spray dried [27]. Depending on the duration of milling, superdisintegrant particles can exist as a binary mixture of colloidal and micron-sized particles [8,27]. The stabilizing action of colloidal superdisintegrant particles was discussed extensively in Azad et al. [212], and will not be covered here. The aforementioned studies [8,27] and recent work [87,242] have demonstrated that (i) wet-milled CCS/SSG at 10% w.r.t. drug allows for replacement of an anionic surfactant (SDS) without significant deterioration in drug release rate and drug loading; (ii) even at 10% w.r.t. drug, they are superior to Mannitol at 100%, a commonly used WSD; (iii) the positive impact of the superdisintegrants correlated positively with their swelling capacity, thus signifying a swelling-induced erosion/disintegration as their action mechanism; and (iv) high drug-loaded (>65%), redispersible, fast-dissolving surfactant-free nanocomposites can be prepared with the use of soluble polymer–superdisintegrant combinations.

### 3.4. Impact of Drying Method

**Figure 10:**
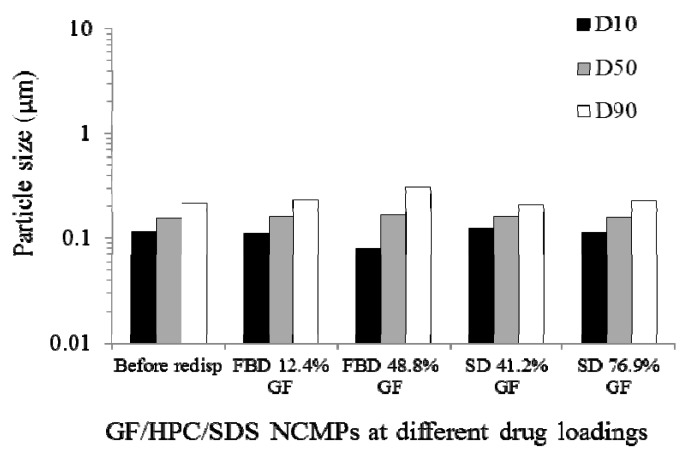
Impact of drug loading in the nanocomposite and drying method on redispersion of griseofulvin (GF)–HPC–SDS nanocomposite microparticles (NCMP) in water after 2 min paddle stirring at 200 rpm. FBD refers to fluid bed coating/drying of the GF nanosuspension onto Pharmatose; SD stands for spray drying. “Before redisp” particle sizes refer to the particle sizes in the wet media milled drug nanosuspension with HPC–SDS. (Adapted from Bhakay et al. [85] with permission from Elsevier, www.elsevier.com).

Eerdenbrugh et al. [34] compared freeze drying and spray drying of 9 model compounds and concluded that dissolution performance was dependent on the drug and its formulation, and that the drying method did not have a significant impact. Bhakay et al. [85] compared redispersion and dissolution performance of griseofulvin and azodicarbonamide nanocomposite particles formed by spray drying versus fluid bed coating/drying. They concluded that both drying methods yielded nanocomposite particles that can be rapidly redispersed into nanoparticles when polymer–surfactant (HPC–SDS) combination was used as stabilizers/dispersants (Figure 10). Sievens et al. [79] wet-cast and dried griseofulvin nanosuspensions to prepare redispersible strip films. The formulation of griseofulvin nanosuspension used by Sievens et al. [79] was very similar that used by Bhakay et al. [8], which allows a comparison of fluid bed coating vs. wet film casting–drying. All these findings signify that the impact of different drying methods on redispersibility–drug dissolution appear to be less than that of the drug–dispersant formulation. To put it differently, redispersible, fast-dissolving nanocomposites can be prepared using any drying method by judicious selection of dispersants’ type/concentration. However, the selection of a drying process is still important to downstream processability of the dried intermediate and scalability, cost, manufacturability, etc., which will be discussed in Section 4. Typical drug loadings in nanocomposite particles formed by fluid bed coating are 10–50% *w*/*w*, whereas higher drug loadings, i.e., as high as ~90% *w*/*w*, can be obtained via freeze and spray drying. Knieke et al. [152] and Bhakay et al. [85] have produced nanocomposite particles with 20% and 48% drug loadings, respectively, by fluid bed coating. In both studies, at higher drug loadings, agglomeration of the coated carrier particles occurred, but the nanocomposites still redispersed back into drug nanoparticles. Even though the fluid bed coating/drying process could form some granules, the combination of polymer–surfactant as dispersants prevented the formation of drug nanoparticle aggregation in the granules, and allowed complete redispersion, as seen in Figure 10. Spray-dried powders, even with 77% drug loading, also released drug nanoparticles without aggregation in the redispersion test (Figure 10).

### 3.5. Incorporating Nanocomposite Intermediate into Final Oral Solid Dosage Forms

Compared to the voluminous information available on the production of drug nanosuspensions and their drying into nanocomposites, relatively scant information is available on the downstream processing of the nanocomposites into final solid dosages such as tablets, capsules, sachets, etc. [55]. In this section, we review some of the representative publications. Basa et al. [65] studied the development of a tablet formulation containing ketoconazole nanoparticles. Ketoconazole nanosuspension stabilized with HPMC–SDS was produced using a wet media mill and fluid bed coated onto lactose monohydrate carrier particles. The layered particles were compressed into tablets, keeping the formulation’s physical properties and dimensional characteristics similar to a marketed tablet formulation. The results showed that the nanocomposites were redispersible, and their tablet exhibited faster dissolution than a marketed tablet. However, drug release from tablets was significantly slower than that from nanosuspensions, which signifies the importance of tabletting formulation–process parameters. Kumar et al. [192] wet media milled lurasidone hydrochloride with HPMC E3–polysorbate and Poloxamer F188 separately, and sprayed the resulting nanosuspensions on mannitol–microcrystalline cellulose into a fluid bed to prepare granules. The granules were compressed, along with tabletting excipients, into an orally disintegrating tablet with <30 s disintegration time; this was shown to significantly enhance drug release compared to a tablet of the as-received drug of identical composition.

Nekkanti et al. [209] prepared a nanosuspension by WMM of candesartan with HPMC–SDS as stabilizers and spray dried the nanosuspension following the addition of mannitol to prepare nanocomposite particles. The spray-dried powder was then directly compressed into tablets, followed by blending with various tabletting excipients. The results showed that the spray-dried nanocomposites were redispersible, and their tablets showed significantly faster dissolution than tablets with a micronized drug of identical formulation and a commercial tablet formulation. Dolenc et al. [243] prepared a celecoxib nanosuspension using PVP–SDS as stabilizer via the emulsion-diffusion method, spray-dried the nanosuspension, blended the dried powder with microcrystalline cellulose, and compressed it into tablets. Tablets with celecoxib nanoparticles dissolved significantly faster than those with micron-sized celecoxib particles.

Tuomela et al. [123] performed a formulation optimization study on tableting of freeze-dried nanosuspensions of itraconazole and indomethacin that were prepared by WMM and stabilized with Poloxamer F127 and Poloxamer F68, respectively. Freeze-dried powders (nanocomposite particles) of both drugs were compressed into tablets via direct compression and wet granulation–compression using various excipients for granulation and tabletting. All the nanocomposite powders dissolved immediately, while the dissolution was slower from tablets; tablet dissolution rates decreased with an increase in the loading of nanocomposite particles in tablets. As the nanocomposite particle loading in tablets increased, more nanoparticle contact was established, which led to a decrease in the porosity of the tablets. Due to decreased porosity, the dissolution medium cannot enter the dense structure, causing longer disintegration and slower dissolution. Hence, an optimum loading of nanocomposite particles in tablets must be used to obtain tablets with suitable strength and dissolution properties, while the advantages of large surface areas of nanoparticles are retained. For both drugs, an optimum concentration of 40% *w*/*w* nanocomposite particles in tablets gave favorable dissolution results. Mauludin et al. [244] prepared a rutin nanosuspenion with SDS as a stabilizer via HPH and freeze-dried it. The powder was blended with standard tabletting excipients and then compressed into tablets. The results showed that the drug release rate from the rutin-nanocrystal loaded tablet was faster than those of a rutin-microcrystal loaded tablet and a marketed tablet.

Besides tablets, drug nanoparticle-laden strip films have been developed recently. Sievens et al. [79], Susarla et al. [131], and Krull et al. [81,82,83], have performed extensive formulation and process optimization studies to manufacture redispersible strip films. The drug release rate is slower in strip films than in the precursor drug nanosuspensions, as the polymeric film provides a diffusion barrier while it swells–erodes–dissolves. Krull et al. [83] investigated the impact of drug loading on film properties and the redispersion/dissolution of griseofulvin nanoparticles. Approximately 40% *w*/*w* griseofulvin (GF) loading was found to be the upper limit that yields films with acceptable mechanical and dissolution properties. Drug loadings of 50 wt.% and 73 wt.% were achieved in HPMC E15 and E4M films, respectively; however, films with drug loadings above 40–50 wt.% were unacceptably brittle. At drug loadings of 10–30 wt.%, redispersion and dissolution properties of the GF films were good. Poor redispersion and significantly slower GF release were observed at drug loadings above 50 wt.%. These results suggest that the greatest barriers to producing pharmaceutical films with high loadings of poorly water-soluble drug nanoparticles are overcoming poor film mechanical properties, and ensuring the recovery of the embedded drug nanoparticles, both of which can be conceivably overcome by further formulation development.

## 4. Some Engineering Considerations for Rational Selection of a Drying Process

For early development/formulation screening and small/bench scale production of nanocomposites, any of the drying processes in Section 3 can be used. The studies reported in Table 2 used small/lab-scale drying equipment. Figure 5 shows that 45% of the studies used freeze drying and 25% used spray drying, which can be explained by their material-sparing nature and cheaper/easier access to small-scale equipment. The popularity of freeze drying and spray drying was also indicated in a previous review paper (Chin et al. [55]). While fluid bed drying (coating or granulation) and nanoextrusion can also be used, they will likely use more material/drug for formulation development. We see a diverging trend between the published, small-scale work and industrial practice in large-scale development and manufacturing; there is no marketed product with freeze-drying. Triglide^®^ was produced using spray drying, and Emend^®^, Tricor^®^ and Rapamune^®^ were produced using fluid bed coating/drying, wet granulation, and tablet coating, respectively. Chin et al. [55] argues that one major reason for the disagreement between academic work and industrial practice, especially at large/commercial scales, is the poor flowability and low bulk density of the spray-dried and freeze-dried products, which demand further unit operations to obtain suitable material characteristics for compression. However, we assert that there are many factors that can influence the selection of a drying process, especially for late-stage development and manufacturing. These factors include operational/engineering considerations such as cost, scalability, cycle time, existing equipment, and available capacity, as well as the prevalent culture in companies, which received much less attention in a unified perspective in pharmaceutical literature.

Freeze-drying is a promising technique for temperature sensitive or thermally labile drugs, and is ideally suited for vaccines and biologics. When used for drying drug nanosuspensions, the resulting powders are porous, which allows for fast disintegration and redispersion [41]. On the other hand, the product is usually in the form of a cake or lumpy powder that needs to be milled/sieved to obtain a powder. Another disadvantage of such powders is the poor flowability and low bulk density, which requires further unit operations such as granulation and addition of a considerable amount of excipients to achieve suitable material characteristics for compression [55,245]. Most importantly, long processing/cycle times, i.e., on the order of day(s), as well as high cost and energy consumption [196,246], render freeze drying unfavorable for commercial-scale production for drying nanosuspensions of small molecule drugs [55,208,246]. Based on all these considerations, we maintain that freeze drying will not be of choice for large-scale development and manufacturing, unless the drug is thermally labile or extremely temperature/shear sensitive.

Spray drying is preferred over freeze-drying, especially for large-scale development and manufacturing, because it is an inherently continuous one-step process that consumes less time and energy [243,247]. In general, spray drying yields powders with rather low densities and poor flowability [55], which requires additional processing steps, such as roller compaction to obtain tablettable intermediate [248]. These challenges can be managed with appropriate downstream processing steps and the use of tabletting excipients; however, some operational and commercial aspects must be considered before process selection. The feed liquid to a spray drier can be an aqueous drug nanosuspension (top down methods) or nanoparticles in water–solvent mixture (e.g., antisolvent precipitation). Spray drying of aqueous nanosuspensions is less challenging, as air can be used as drying medium, the residual solvent is harmless, and water vapor can be exhausted into the atmosphere directly, whereas spray drying of suspensions with organic solvents requires direct integration of the anti-solvent precipitation equipment with the spray drier (see e.g., [62,141]) and the use of nitrogen or other inert gases, along with specialized solvent recovery systems. It must be noted that large-scale spray driers require specialized facilities, and there are few available pharmaceutical spray drying facilities in the market for product and process development purposes [249]. Hence, the selection of spray drying entails consideration of available plants (in-house vs. CMOs) with appropriate equipment, available capacity, and potential new investment in expertise, installation, and operation of capital-intensive large-scale spray driers and facilities.

Fluid bed coating of a drug nanosuspension onto inert beads such as sugar, cellulose, or other inert excipients, i.e., bead-layering, appears to have several advantages, such good flowability and high bulk density of the coated powders compared with freeze-dried and spray-dried powders. As a result, downstream processing of these powders, such as direct filling into capsules/sachets or compressing them into tablets with additional excipients, is simpler. In addition, fluid bed coating is a versatile process and can also be used to apply a second functional coating onto the beads, which could, for example, afford enteric or mucoadhesive properties [64,248]. Although fluid bed coating has certain disadvantages such as limited drug load achievable and the rather long coating time [250], it has already been used for several oral dosage forms on the market, including Emend. It is also possible to granulate excipients using the drug nanosuspension as “binder solution”, with additional binder polymer if needed, and to produce a nanocomposite in the form of granulated powder, which also has good flowability and high bulk density [192]. Fluid bed granulation is much faster than fluid bed coating because high spray rates can be used to reduce cycle time, which favors particle agglomeration as a desirable transformation unlike in coating. From an operational/engineering perspective, fluid bed processing is the best available option to most pharmaceutical companies, as fluid bed drying is a standard pharmaceutical unit operation as part of wet granulation process train, and even traditional fluid bed driers can easily be retrofitted to perform coating/granulation, i.e., with the addition of spray guns, without major capital investment.

Spray drying and fluid bed coating/granulation/drying processes entail atomization of the feed drug nanosuspensions into droplets. Generating small droplets during atomization puts a limit on the viscosity; highly viscous suspensions that have high solids loading (drug nanoparticles and dissolved species) and/or high MW polymers or swellable dispersants cannot be processed without dilution. Practically, certain polymer types and MWs are not conducive to these spraying processes, which restrict the choice of polymers/polymer grades. Being a continuous process and having the capability to handle viscous polymers, a recently-developed nanoextrusion process could be advantageous for drying drug nanosuspensions [75,76,78]. The product, in the form of extrudates, is dry milled into a powder, and the powder can be blended with excipients for tabletting. For the nanoextrusion process to be thermally efficient and the extrudates to have sufficiently low moisture content, the solids loading in the feed must be maximized. Drug nanosuspensions prepared with media milling can have up to ~50% drug loading, whereas bottom-up methods tend to produce dilute nanosuspensions (<10% drug loading) [23]. Hence, drug nanosuspensions may need to be filtered prior to nanoextrusion, and this aspect, as well as downstream processing of the extrudates into tablets, warrants further investigation. 

Wet film casting–drying of a precursor drug–polymer suspension, which is prepared by mixing a drug nanosuspension with a film-forming polymer–plasticizer solution, yields drug nanoparticle-laden polymer strip films. This relatively new continuous drying method has shown itself to be promising [81,82,83], and, like the nanoextrusion process, it can handle suspensions that may not be effectively atomized without dilution. Unlike all the previously mentioned drying methods that eventually produce a nanocomposite powder/extrudate, this method yields a completely different dosage format, i.e., ~50–200 μm strip films that disintegrate and release the drug rapidly in the oral cavity. Such films can be taken with little to no water, and thus, have significant advantages over tablets regarding patient compliance for the geriatric–pediatric–dysphagic population. 

## 5. Additional Insights into Development of Redispersible, Fast-Dissolving Nanocomposites

Unlike for BCS Classes I and III drugs (high solubility), the ideal drug release for poorly water-soluble drugs (BCS Class II drugs) from solid oral dosages containing nanocomposites is generally the one that corresponds to the fastest drug release in vitro, because the low drug solubility and ensuing slow dissolution of such drugs is the rate limiting step in absorption through the gastrointestinal tract. It is well-known that solid oral dosages containing drug-laden nanocomposites of poorly water-soluble drugs exhibit significantly higher drug bioavailability and exposure in in vivo testing on animals and/or clinical testing on humans compared with dosages containing the as-received micron-sized drug particles [113,209,251,252,253]. Hence, the major formulation criterion for developing nanocomposites incorporating poorly water-soluble drug nanoparticles and final solid dosages containing drug-laden nanocomposites has been to attain significantly faster in vitro drug dissolution than that from as-received micron-sized drug particles, their physical mixtures with the excipients of the nanocomposites, and finally, the same oral solid dosages containing as-received micron-sized drug particles (e.g., [66,85,86,87]). In fact, the use of this criterion has already been illustrated with several examples throughout this review paper, especially in Section 3.5, for final oral dosages prepared with various drying methods. Ideally, the fastest dissolution profile with drug nanoparticles is attained upon dosing a nanosuspension, which is not a desirable dosage form from a patient preference/compliance perspective. Unlike drug release from nanosuspensions, drug release from nanocomposites or final solid dosage forms involves the rate-limiting step of nanoparticle recovery during in vitro or in vivo redispersion–dissolution; therefore, the drug release is slower from nanocomposites than nanosuspension. A comparative assessment of in vitro drug release from nanocomposites (dried nanosuspension)/final solid dosages, with respect to that from a nanosuspension, may be used to gauge the effectiveness of the dissolution rate enhancement upon the use of nanocomposites. Considering that some of the biggest challenges in dissolution rate enhancement originate from poor and slow redispersion of the nanocomposites leading to slow recovery of drug nanoparticles [66,92], redispersible drug nanocomposite formulations offer a promising solution, as discussed below.

Section 3 summarized findings of various studies which examine the impact of various classes of dispersants on redispersion–drug release from the nanocomposites. It should be noted that there is no “universal formulation approach” that allows for proper stabilization of all drug nanosuspensions and redispersible, fast drug release from the nanocomposites. Chemical structure, solubility, and hydrophobicity/log*P* of drugs are different, which need different wetting agents and stabilizers to stabilize the drug nanoparticles [23]. Moreover, aggregation of drug nanoparticles during drying is a complex thermodynamic–kinetic phenomenon that cannot be solely predicted based on the properties of the drugs or drug–dispersant pairs. For example, it is well-known that transport processes such as drying rate and diffusion rate of dispersants during the evaporation [89], as well changing viscosity of the suspensions during drying, could have an impact on the nanoparticle aggregation and redispersibility [74].

Eerdenbrugh et al. [34] attempted to correlate the dissolution of 9 wet media milled poorly water-soluble drugs namely cinnarizine, griseofulvin, indomethacin, itraconazole, loviride, mebendazole, naproxen, phenylbutazone and phenytoin, with their log*P* alone. TPGS was used as the only stabilizer/dispersant. All nanosuspensions were spray-dried and compared for dissolution performance, taking the dissolution of non-nanosized drug product as basis of comparison. The SLS concentration in the aqueous dissolution medium, corresponding to identical drug compound solubility of 0.375 mg/mL, was determined from the linear fit of the concentration of the solubilized compound as a function of SLS concentration, taking into account the 0.5, 1 and 2% (*w*/*v*) SLS data points. Keeping the same drug solubiliy in the dissolution medium, the impact of drug log*P* was investigated. The results show that drugs with high log*P* values such as phenylbutazone (4.2), itraconazole (8.5), and cinnarizine (6.1) exhibited slower dissolution. For indomethacin (3.3), loviride (3.7), and phenytoin (2.3), dissolution was not compromised upon drying of the drug nanosuspensions. The more hydrophobic drugs were presumed to form agglomerates (irreversible aggregates) during drying, which reduced the dissolution rate. Hence, Eerdenbrugh et al. [34] suggest that for such drugs, additional matrix formers (dispersants) are required to ensure rapid dissolution. While this study appears to be unique in the literature, providing excellent “general guidance” about drugs with high log*P*, several caveats must be noted. First, for griseofulvin (2.2), mebendazole (3.3), and naproxen (3.0), the dissolution results were inconclusive due to poor discrimination between nanosized and non-nanosized products. Despite having the lowest log*P* in all drugs studied in ref. [34], griseofulvin nanosuspensions require a soluble polymer–surfactant combination to prevent aggregate formation during drying [85,86]. Second, to keep identical solubility in the dissolution medium, Eerdenbrugh et al. [34] varied the SLS concentration in the dissolution medium, which could have affected the drug wettability and drug release during dissolution. Such an effect could confound results vis a vis the role of log*P* alone on the formation of agglomerates and its impact on the dissolution. In fact, it is speculated that just the mere presence of SLS and ensuing improved drug wettability of the nanocomposites could have positively impacted the dissolution rate. Such an impact of SLS in the redispersion–dissolution medium on the redispersion of drug-laden nanocomposites and drug release has been demonstrated elsewhere [66,86]. Finally, actual redispersion of the nanocomposites was not studied in ref. [34], which could have shed more light on the dissolution results. Even Eerdenbrugh et al. claimed that log*P* can be used as a quick, but “rough” prediction tool. It is clear that further research is needed to understand the impact of drug properties before general conclusions can be drawn as to whether just a surfactant, like TPGS, or a polymer alone would suffice, or whether additional dispersants are required to prevent the aggregation of a given drug.

**Figure 11:**
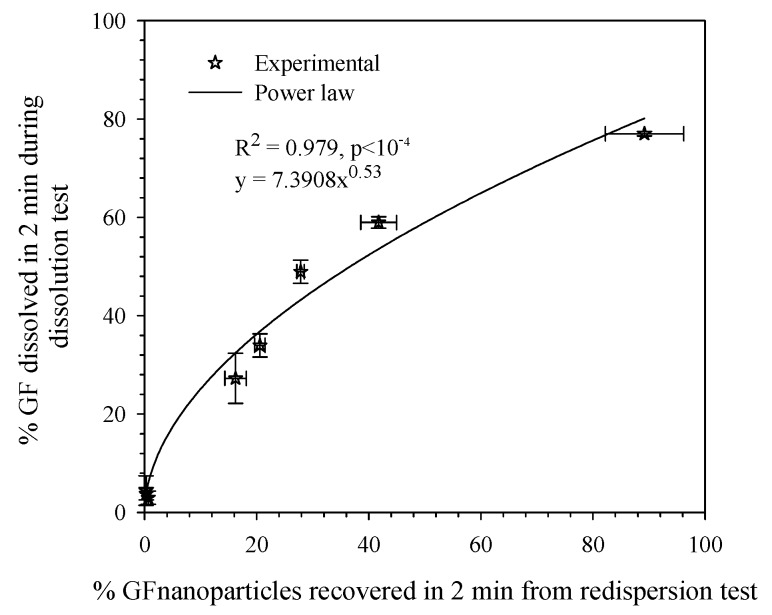
A correlation between percentage of GF dissolved in 2 min in the USP II dissolution test and percentage of GF nanoparticles recovered in 2 min during the agitated redispersion test with a paddle stirrer. Markers correspond to various dispersant formulations in the GF nanocomposites prepared by fluidized bed coating of wet media milled GF suspensions on Pharmatose. The redispersion–dissolution medium is water. (Adapted from Bhakay et al. [8] with permission from Taylor & Francis Ltd., www.tandfonline.com.

Our analysis of Table 2 reveals that 73% (88/120) of the formulations were subjected to redispersion testing, and 63/120 formulations were subjected to both redispersion and dissolution testing. Out of 63 formulations, 60 formulations that exhibited the best redispersibility also exhibited the best dissolution performance. This finding is not surprising, as redispersion and drug nanoparticle recovery is the preliminary step in drug release from drug-laden nanocomposites; redispersion testing could be used to make inferences on the formation of irreversible aggregates during drying. Also, as indicated in Section 3.2.4, during 2012–2017, there is a resurgence of interest in redispersion phenomena: 73% vs. 40% [55] of the formulations reported were subjected to redispersion. Hence, both quiescent and agitated redispersion tests can be used to “predict” fast drug dissolution and rank-order dispersant formulations [8,66,92]. In fact, the relationship between redispersion and dissolution can be quantitative, as demonstrated in Figure 11 [8]. A good correlation between griseofulvin (GF) released in 2 min from drug nanocomposites with various dispersant formulations during a dissolution test and percentage of nanoparticles released during an agitated redispersion test was obtained. Similar correlations were obtained between dissolution test results and measured turbidity in a quiescent redispersion test [92].

Analysis of the redispersible formulations in Table 2 allows us to assess which dispersant classes are widely used in such formulations (refer to Figure 8). Soluble polymers and surfactants are the most commonly used dispersants, whereas water-insoluble dispersants and crosslinked polymers were used in fewer formulations. Most redispersible formulations have a combination of different dispersant classes; polymers alone (12%) or surfactants alone (8%) are not as widely used. Polymer–surfactant are the most used dispersants in redispersible formulations, followed by polymer–surfactant–WSD (mostly sugar/sugar alcohols). This finding is not surprising, because the use of multiple classes of dispersants could have a synergistic or additive impact on prevention of aggregation in the nanosuspensions during preparation/storage and during drying, as discussed in Section 3. As a novel class of dispersants in drug-laden nanocomposites, crosslinked polymers such as wet-milled superdisintegrants have recently been shown to be superior to WSDs like mannitol and sucrose in the preparation of surfactant-free nanocomposites. Considering that higher dispersant concentrations usually favor redispersion and drug release at the expense of drug loading, a formulation optimization must be performed following initial screening of polymer–surfactant or polymer–surfactant–WSD. In the screening, various quiescent or agitated redispersion methods can be used, because those formulations that do not redisperse in water or other biorelevant fluids will most likely lead to inferior dissolution enhancement.

## 6. Concluding Remarks and Future Research Directions

This comprehensive review paper provides a holistic view of drug nanosuspension and nanocomposite formation methods, selection of dispersants based on their functionalities in view of the statistical analysis of 92 publications from 2012–2017, characterization methods, and novel approaches in formulation and process development. The statistical analysis shows that wet media milling is the most commonly-used method for the preparation of drug nanosuspensions in both academic studies and industrial practice/marketed products. On the other hand, we note a divergence of the choice of a drying method in academia vs. industry for the preparation of drug-laden nanocomposites, which can be explained by the development scale, operational/engineering considerations such as cost, scalability, cycle time, existing equipment and available capacity as well as the prevalent culture in companies, which has received much less attention in the pharmaceutical literature. Hence, the selection of a drying method should take these factors into account, as well as the intended final dosage form and delivery route, physico-chemical properties, and degradation characteristics of the drug, and its target patient population. 

The following general guidance and considerations for robust formulation development of fast-dissolving nanocomposites emerge from this review: (i) preparation of stabilized, aggregate-free nanosuspensions is necessary, but insufficient for significant dissolution enhancement; (ii) the type–concentration of dispersants must be selected to ensure the stability of the nanosuspensions prior to drying, as well as to minimize aggregation during the drying and fast recovery of the primary drug nanoparticles during dissolution; (iii) in general, higher dispersant concentration favors both drug nanosuspension stabilization, except for some surfactants, and faster redispersion–drug release at the expense of reduced drug payload in the nanocomposites; (iv) thus, usage of dispersants must be optimized to ensure sufficiently high drug payload; (v) the combined use of different types of dispersants with various functionalities, especially soluble polymer–surfactant, soluble polymer–surfactant–WSD or soluble polymer–superdisintegrant (for surfactant-free formulations), may help to enhance nanosuspension stabilization and dissolution, and could even bring synergistic improvements; and (vi) analysis of drug log*P* and drug wettability enhancement by dispersants, as well as redispersion tests, could help in selecting robust redispersible formulations. This review paper has also demonstrated the criticality of the redispersion phenomenon and the use of various agitated–quiescent redispersion tests, in addition to standardized dissolution tests, for robust and streamlined development of redispersible, fast-dissolving drug-laden nanocomposites. The use of redispersion testing has increased recently, and it is expected that such tests will be used as commonly as dissolution tests in nanoformulation development.

Future research should be directed to a more fundamental understanding of aggregate formation during nanosuspension drying, including capillary pressure mechanism and nanoparticle phase separation phenomena, impact of drug properties, as well as drug–dispersant interactions for both nanosuspension stabilization and drying, and development of “standardized” redispersion tests for the screening of various nanocomposite formulations. This review also points to a significant need for more research on the incorporation of the nanocomposites in standard oral solid dosage forms, such as tablets and capsules, as well as on relatively new oral solid dosages, such as drug nanoparticle-laden polymeric strip films and extrudate-based products.

## Figures and Tables

**Table 1:** Drug nanoparticle-based marketed products approved by FDA (Adapted from Malamatari et al. [31] with permission from Elsevier, www.elsevier.com).

Product Name/Company	Drug	Nanoparticle Preparation Method ^a^	Final Dosage	Year Approved
Avinza^®^/King Pharma	Morphine sulfate	WMM	Capsule	2002
Azopt^®^/Alcon	Brinzolmid	WMM	Suspension	1998
Cesamet^®^/Lilly	Nabilon	Precipitation	Capsule	2005
Emend^®^/Merck	Aprepitant	WMM	Capsule	2003
Focalin XR^®^/Novartis	Dexmethylphenidate HCl	WMM	Capsule	2001
Gris-Peg^®^/Novartis	Griseofulvin	Precipitation	Tablet	1982
Herbesser^®^/Mitsubishi	Diltiazem	WMM	Tablet	2002
Invega Sustenna^®^/Johnson & Johnson	Paliperidone palmitate	WMM	Suspension	2009
Megace ES^®^/Par Pharmaceutical	Megestrol acetate	WMM	Suspension	2005
Neprelan^®^/Wyeth	Naproxen sodium	WMM	Tablet	2006
Rapamune^®^/Wyeth	Sirolimus (rapamycin)	WMM	Suspension, Tablet	2000
Ritalin LA^®^/Novartis	Methylphenidate HCl	WMM	Capsule	2002
Theodur^®^/Mitsubishi Tanabe Pharma	Theophylline	WMM	Tablet, Capsule	2008
Tricor^®^/Abbott	Fenofibrate	WMM	Tablet	2004
Triglide^®^/SkyePharma	Fenofibrate	HPH	Tablet	2005
Verelan PM^®^/Schwarz Pharma	Verapamil HCl	WMM	Capsule	1998
Zanaflex^®^/Acorda	Tizanidine HCl	WMM	Capsule	2002

^a^ HPH: High-pressure homogenization; WMM: Wet media milling.

**Table 2:** Review of recent literature (2012–2017) regarding the preparation methods used for drug nanosuspension preparation, drying methods used for converting nanosuspensions into nanocomposites, dispersants used in the formulations, and redispersion methods used for nanoparticle recovery.

Method for Drug Nanosuspension Preparation	Drying Method	Drug and Its Assay in Nanocomposites (% *w*/*w*)	Dispersants in Nanocomposites ^a,b^	Redispersion Method (If Used)	*d*_50_^c^, dvm ^d^, Cumulant ^e^ Size Before and After Redispersion (µm)		Reference
					Before	After	
LASP–Ultrasonication	Freeze drying	Carvedilol (–)	(Alpha tocopherol succinate, SDS, Maltose)	_	0.212	_^f^	Liu et al., (2012) [108]
WMM	Freeze drying	Naproxen (–)	(HPC, PEG Carrageenan), Sucrose	Dried powders were dispersed in 150 mL water and sonicated for 1 min	0.148 ^d^	0.150 ^d,g^	Chung et al., (2012) [72]
WMM	Freeze drying	Model drug (–)	(Poloxamer 338, PVP K15), Cremophor EL Sucrose, Trehalose	An aqueous solution of 5 mg/mL poloxamer 338 was used as a medium	_	0.165 ^g^	Beirowski et al., (2012) [71]
WMM	Wet film casting–drying	Griseofulvin (3.8 mg/cm^2^) Naproxen (3.3 mg/cm^2^) Fenofibrate (4.8 mg/cm^2^)	(HPMC E15LV, SDS, Glycerin)	0.71 cm^2^ circular films were put in 15 mL water and stirred for 10 min via magnetic stirrer	0.163 0.144 0.207	0.175 ^g^ 0.145 ^g^ 0.256 ^f^	Sievens et al., (2012) [79]
WMM	Electrospray drying	Naproxen (–)	(HPC)	Dried powders were placed in 150 mL water and sonicated for 4 min	~0.110 ^d^	0.100 ^d,g^	Ho and Lee (2012) [120]
WMM	Fluid bed granulation/drying	Compound A (9.19%)	(Vitamin E TPGS, HPMC 3, Mannitol DC), Lactose monohydrate	_	~0.220	_ ^f^	Bose et al., (2012) [130]
WMM	Fluid bed coating/drying	Griseofulvin (12.4%)	(HPC SL, SDS), Mannitol, Pharmatose (core)	1 g dried sample was dispersed in 30 mL water for 2 min using paddle stirring (200 rpm), pipette stirring, magnetic stirring (100 rpm) and sonication	0.145	0.150 ^f^	Bhakay et al., (2013) [66]
LASP–Ultrasonication	Wet film casting–drying	Griseofulvin (3.95%)	(HPMC E15LV, HPMC E4M, Pluronic F127, Glycerin)	Dried films were dispersed in water	0.580	~2.000 ^f^	Beck et al., (2013) [109]
WMM	Spray drying	Miconazole (45%) Itraconazole (44%)	(HPC, SDS, Mannitol), MCC	Dried samples were dispersed in water and shaken manually	0.157 ^d^ 0.144 ^d^	~0.200 ^d,f^ ~0.150 ^d,f^	Cerdeira et al., (2013) [121]
	Freeze drying	Miconazole (47%) Itraconazole (42%)	(HPC, SDS, Mannitol), MCC	Dried samples were dispersed in water and shaken manually	0.182 ^d^ 0.192 ^d^	~0.198 ^d,f^ ~0.200 ^d^	
WMM	Wet film casting–drying	Griseofulvin (1.87 mg/cm^2^)	(SDS, HPMC E15, Glycerin)	0.715 cm^2^ circular films were put in 15 mL water and stirred for 10 min using magnetic stirrer	0.163	0.164 ^f^	Susarla et al., (2013) [131]
WMM	Spray-freeze drying	Phenytoin (–)	(PVP, SLS)	Powders equivalent to 2 mg phenytoin were dispersed in 10 mL of dissolution media (pH 1.2 and 6.8) and stirred up to 30 min via magnetic stirrer	0.170–0.180	~0.400 ^f^	Niwa et al., (2013) [7]
LASP	Freeze drying	Curcumin (37.6%)	(PEG-PLA, PVP BP, HPBCD)	Dried samples were dispersed in DI-water	0.055 ^e^	0.076 ^e,g^	Cheng et al., (2013) [132]
LASP–Ultrasonication	Fluid bed coating/drying	Indomethacin (–)	(β-lactoglobulin, PVP K30, Trehalose), Nonpareil (core), Soybean Protein Isolate, Whey protein isolate	100 mg dried product was dispersed in 10 mL DI-water via manual shaking for 1 min	0.243 ^e^	0.289 ^e,f^	He et al., (2013) [133]
Acid-base neutralization	Spray drying	Diosmin (–)	(HPMC, Mannitol), MC	Dried powders were dispersed in distilled water	0.336 ^e^	0.316 ^e,f^	Freag et al., (2013) [110]
LASP–HPH	Freeze drying	Nimodipine (–)	(Poloxamer 407, Sodium deoxycholate, HPMC E5, Mannitol, Maltose)	Beckmann Coulter LS 230	0.159 ^d^	0.148 ^d,f^	Fu et al., (2013) [134]
Ultrasonication	Freeze drying	Fenofibrate (–)	(Poloxamer 188, Mannitol), PVP K25, Poloxamer 407, SDS, Tween 80	_	0.460 ^e^	_ ^f^	Ige et al., (2013) [135]
WMM	Spray drying	Fenofibrate (–)	(HPMC E5, SDS, Mannitol), Sucrose, Glucose, Maltose, Lactose	20 mg dry powder was added in 5 mL of DI water and shaken manually	0.452	0.499 ^f^	Zuo et al., (2013) [136]
Ultrasonication	Nanoextrusion	TiO_2_ (–)	(Citric acid monohydrate, SDS, Soluplus), Tween60, Cremophor EL, Cremophor RH 40	_	~0.182 ^e^	_ ^g^	Khinast et al., (2013) [75]
HPH	Wet film casting–drying	Herpetrione (10 mg/4 cm^2^)	(PVP K30, SDS, L-HPC, HPMC E50, MCC, PEG 400, Mannitol)	2 × 2 cm^2^ film was placed into distilled water and manually shaken for 30 s	0.260 ^e^	0.280 ^e,f^	Shen et al., (2013) [106]
WMM	Freeze drying	Curcumin didecanoate (–)	(Poloxamer 188)	2 mg powder was suspended in peanut oil and sonicated for 1 min	~0.500 ^e^	0.517 ^e,g^	Wei et al., (2013) [137]
WMM	Spray drying	Naproxen (–) Indomethacin (–)	(HPMC E15), Dowfax 2A 1(Dowfax 2A1), HPMC E15	Powders were suspended in saturated and filtered solution of the drug in 30% glycerin solution	0.309 ^e^ 0.223 ^e^	0.400 ^e,f^ 0.351 ^e,f^	Kumar et al., (2014a) [138]
WMM	Spray drying	Indomethacin (–)	(Dowfax 2A1, Maltose), Trehalose, Lactose, Mannitol, Ficoll PM70, Maltodextrin	Powders were suspended in saturated and filtered solution of indomethacin in 30% glycerin solution	0.200–0.300 ^e^	0.179 ^e,g^	Kumar et al., (2014b) [139]
	Freeze drying	Indomethacin (–)	(Dowfax 2A1, Sucrose), Trehalose, Lactose, Mannitol, Ficoll PM70, Maltodextrin		0.197 ^e^	0.208 ^e,g^	
HPH	Freeze drying	Simvastatin (–)	(Soya Lecithin, Mannitol)	_	0.316 ^e^	_ ^f^	Asma et al., (2014) [140]
WMM	Nanoextrusion	Phenytoin (–)	(Tween 80, Soluplus), Tween 20, Kolliphor P188, Kollicoat IR	_	0.335 ^e^	_ ^f^	Baumgartner et al., (2014) [76]
LASP	Spray drying	Fenofibrate (–)	(PVP 10, MMT), Lactose	_	<1.00 ^e^	_ ^f^	Dong et al., (2014) [141]
WMM	Freeze drying	Efavirenz (–)	(PVP K30, SLS, Trehalose), Mannitol, Poloxamer 188 and 407	_	0.320 ^e^	_ ^f^	Patel et al., (2014) [142]
HPH	Spray drying	Lovastatin (–)	(PVP K17, SDS), HPMC 2910, Polyvinyl alcohol, PVP K30 and K12, Poloxamer 188 and 407	Dried powders were dispersed in distilled water and shaken manually for 10 s RDI = mean redispersion size/nanosuspesion size ×100	0.380 ^e^	110% ^f^	Zhang et al., (2014) [26]
LASP–Ultrasonication	Freeze drying	Tadalafil (–)	(Tween 80, Span80), SLS PEG 4000, PVP K30, Pluronic F-127, Methocel E50 and E5, Span 20 and 60	_	0.193 ^e^	_ ^f^	Obeidat et al., (2014) [143]
LASP–Ultrasonication	Freeze drying	Zaltoprofen (–)	(Poloxamer 188, SLS), Poloxamer 407	Dried samples were diluted with 20 mL distilled water to observe redispersibility	0.179 ^e^	_ ^f^	Papdiwal et al., (2014) [144]
	Freeze drying	Quercetin dehydrate (–)	(Tween 80)	_	~0.430 ^e^	_ ^f^	
LASP	Freeze drying	Celecoxib (–)	(Soluplus)	_	0.293 ^e^	_ ^f^	Homayouni et al., (2014) [145]
LASP–HPH	Freeze drying	Celecoxib (–)	(Soluplus)	_	0.577 ^e^	_ ^f^	
LASP–Ultrasonication–HPH	Freeze drying	Diacerein (–)	(SDS, Mannitol), PVA, Sodium deoxycholate, Sucrose	Malvern Zetasizer^®^ Nano ZS90	0.374 ^e^	0.374 ^e,f^	Elsayed et al., (2014) [146]
WMM	Fluid bed coating/drying	Bifendate (–)	(HPC SL, SLS, Mannitol), MCC (core), HPMC E5, PVP K30, Poloxamer 407 and 188	5 g dried products were dispersed in 100 mL purified water and paddle stirred at 100 rpm for 5 min	0.139 ^d^	0.360 ^d,f^	Yao et al., (2014) [147]
WMM	Freeze drying	Telmisartan (–)	(Poloxamer 188, Trehalose), Poloxamer 407, PVA, PVP K30, SLS, Tween 80, HPMC E5, HPC, Glucose, Lactose, Mannitol, Sucrose	50 mg dried product was dispersed in 5 mL distilled water and sonicated for 15–30 s	~0.335 ^e^	~0.337 ^e,f^	Patel et al., (2014) [148]
LASP	Spray drying	Celecoxib (–)	(PVP K30)	–	0.321 ^e^	_ ^f^	Homayouni et al., (2014) [149]
	Freeze drying	Celecoxib (–)	(PVP K30)	_	0.321 ^e^	_ ^f^	
LASP–HPH	Freeze drying	Celecoxib (–)	(PVP K30)	_	0.450 ^e^	_ ^f^	
LASP–Ultrasonication	Freeze drying	Acyclovir (–)	(Poloxamer 188, PVP K30, Sucrose), TPGS, Tween 80, Mannitol, MCC	Malvern Mastersizer	0.274 ^e^	0.353 ^e,f^	Bhalekar et al., (2014) [150]
WMM	Fluid bed coating/drying	Griseofulvin (48.8%) Azodicarbonamide (40.4%)	(HPC SL, SDS), Pharmatose (core)	Dried product equivalent to 5 wt.% drug (w.r.t. suspension) dispersed in 20 mL water stirred for 2 min using impeller at 200 rpm	0.160 0.250	~0.160 ^f^ ~0.250 ^f^	Bhakay et. al., (2014) [85]
	Spray drying	Griseofulvin (76.9%) Azodicarbonamide (74%)	(HPC SL, SDS), Mannitol		0.160 0.250	~0.160 ^f^ ~0.250 ^f^	
WMM	Spray drying	Itraconazole (–)	(PVP40, SLS), Methocel E3, Methylcellulose A15, HPMC E5, HPMC E15, HPC, PVA, Kollidon 30, PVP40, Poloxamer 188 and 407	Powders were suspended in saturated and filtered solution of indomethacin in 30% glycerin solution	0.283 ^e^	0.310 ^e,f^	Kumar et al., (2015) [151]
WMM	Fluid bed coating/drying	Fenofibrate (14.1%)	(HPMC E3, SDS, Mannitol), Pharmatose (core), GranuLac (core), potato starch (core)	100 mg dried powders were dispersed in 8 mL DI water and shaken manually for 30 s	0.160	0.265 ^f^	Knieke et al., (2015) [152]
WMM	Spray drying	Griseofulvin (75.2%)	(HPC SL, SDS), CCS, SSG, Mannitol	Qualitative assessment: 10 mg dried powder was dispersed in 4 mL DI water and observed the cloudiness	0.161	Cloudy supernatant ^f^	Azad et al., (2015) [27]
WMM	Wet film casting–drying	Fenofibrate (17%) Griseofulvin (14.5%) Naproxen (14.2%) Phenylbutazone (15.1%) Azodicarbonamide (17.7%)	(SDS, HPMC E15 LV, Glycerin)	~0.71 cm^2^ circular films were dispersed in 3 mL DI water and vortex mixed for 3–5 min	0.178 0.16 10.136 0.176 0.278	0.283 ^f^ 0.164 ^f^ 0.134 ^f^ 0.184 ^f^ 0.352 ^f^	Krull et al., (2015) [153]
WMM	Spray drying	Naproxen (–)	(HPMC E15, Trehalose), Tween 80, Lactose	Powders were suspended in saturated and filtered solution of naproxen in 30% glycerin solution	0.243 ^e^	0.282 ^e,f^	Kumar et al., (2015) [154]
HPH	Freeze drying	Harmine (–)	(HPMC E15, Sorbitol), TPGS, Tween 80, CMS-Na, RH40, Sucrose, Glucose, Trehalose, Mannitol	Malvern Mastersizer RDI = mean redispersion size/nanosuspension size ×100	0.500–0.700	~100% ^g^	Yue et al., (2015) [155]
	Spray drying	Harmine (–)	(CMS-Na), HPMC E15, TPGS, Tween 80, CMS-Na, RH40		0.500–0.700	~100% ^g^	
WMM	Wet film casting–drying	Griseofulvin (15.8%)	(HPMC E15, SDS, Glycerin, CCS/HPMC E4), SSG, CP, GG, XG, Pectin	~0.71 cm^2^ circular films were dispersed in 10 mL DI water and vortex mixed for 1 min	0.160	0.160 ^f^	Susarla et al., (2015) [80]
LASP	Freeze drying	Ursolic acid (–)	(TPGS 1000, Trehalose), Maltose, Glucose, Sucrose, PEG2000	Dried powders were dispersed in water and shaken manually	0.127 ^e^	0.239 ^e,f^	Ge et al., (2015) [156]
WMM	Spray drying	Indomethacin (36.3%)	(Poloxamers 188, Mannitol, L-leucine), Poloxamers 407 and184, TPGS 1000	Malvern Nano ZS	0.263 ^e^	0.417 ^e,f^	Malamatari et al., (2015) [157]
HPH	Spray drying	Itraconazole (–)	(Poloxamers 407, SLS, Mannitol)	Malvern Zetasizer	~0.316 ^e^	~0.320 ^e,f^	Sun et al., (2015) [107]
LASP–Ultrasonication	Freeze drying	Naproxen (9.42%)	(HPMC)	_	0.530 ^e^	_ ^f^	Mishra et al., (2015) [158]
HPH	Wet film casting–drying	Quercetin (10 mg/6 cm^2^)	(Maltodextrins, Glycerin, Tween 80, Span 80)	Malvern Zetasizer^®^ Nano ZS	0.753 ^e^	0.781 ^e,f^	Lai et al., (2015) [159]
	Freeze drying	Quercetin (–)	(Tween 80)		0.753 ^e^	~0.921 ^e,f^	
LASP–Ultrasonication	Freeze drying	Teniposide (–)	(PVP K30), Poloxamer 188, HPMC	Powders were suspended with 5% glucose and vortexed for 5 s	0.151 ^e^	0.151 ^e,f^	He et al., (2015) [160]
Acid-base neutralization–Ultrasonication	Spray drying	Glipizide (–)	(SLS, MCC), Poloxamer, PVP, HPMC, Tween 80, Mannitol, Sorbitol	_	0.262	_ ^f^	Elham et al., (2015) [111]
ME–HPH	Freeze drying	Lambda-cyhalothrin (–)	(MRES, SDS), PVP K30, PVP K90, SL, Tween 80, PEGNPE, HPMC, Poloxamer 188, Span 80, Mannitol	_	0.016 ^e^	_ ^g^	Pan et al., (2015) [161]
WMM	Wet film casting–drying	Griseofulvin (8.75%)	(Pullulan, SDS, XG, Glycerin)	10 mg of the dry film was dispersed in 20 mL DI water and vortexed for 2 min at 1500 rpm	0.168	0.379	Krull et al., (2016) [162]
WMM	Wet film casting–drying	Griseofulvin (15.4%)	(HPMC E15LV, SDS, PEG 400), Triacetin, Glycerin	~0.7 cm^2^ circular dried film was dispersed in 3 mL DI water and vortexed at 1500 rpm for 3–5 min	0.159	0.169 ^f^	Krull et al., (2016) [81]
ME	Wet film casting–drying	Fenofibrate (5.03%)	(Pluronic F68, HPMC E15 LV, Glycerin)	2.54 cm diameter film was dispersed in 10 mL DI water and stirred for 5 min at 475 rpm using a magnetic stirrer	~0.500	~0.800 ^f^	Bhakay et al., (2016) [40]
WMM	Fluid bed coating/drying	Griseofulvin (12.0%)	(HPC L, SDS), Pharmatose (core), HPC SSL, HPC SL	1 g of the dried powders was dispersed in 30 mL DI water and paddle stirred for 2 min at 200 rpm	~0.250	~0.250 ^f^	Li et al., (2016) [86]
HPH	Spray drying	Itraconazole (–)	(Tween 20, Methocel E5)	Powder corresponding to 0.2 g drug was dispersed in 25 mL water and shaken manually for 30 s	0.313 ^e^	0.425 ^e^	Bonda et al., (2016) [163]
HPH	Spray drying	Albendazole (–)	(Poloxamer 188), SDS, Poloxamer 407	20 mg powders were dispersed in 5 mL DI water and manually shaken for 1 min	0.450 ^e^	0.550 ^e,f^	Parades et al., (2016) [164]
HPH	Nanoextrusion	Efavirenz (1.02%)	(SLS, Kollidon^®^ 30, Soluplus)	Extrudates were dispersed in water and vortexed for 30 s	0.320 ^e^	~0.030 ^f^	Ye et al., (2016) [77]
LASP–HPH	Freeze drying	Ursodeoxycholic (–)	(PVP K30, Sucrose/Glucose), TPGS, Poloxamer 188, Lactose Poloxamer 407, RH40, HPMC, SDS, Tween 80, PEG4000, Trehalose, CMS-Na, MCCS, Mannitol, Sorbitol	Malvern Mastersizer RDI = mean redispersion size/nanosuspension size ×100	0.600–0.900	~100% ^g^	Ma et al., (2016) [165]
LASP	Freeze drying	Gambogenic acid (29.7%)	(PVP K30, PEG2000, Trehalose, Mannitol)	Malvern Zetasizer	~0.184 ^e^	~0.198 ^e,f^	Yuan et al., (2016) [166]
HPH	Spray drying	Model drug (–)	(HPC L, MCCS), HPMC, MCC, CCS, Trehalose, Lactose	Malvern Mastersizer	0.461	0.478 ^f^	Dan et al., (2016) [167]
LASP–Ultrasonication	Freeze drying	20(S)-protopanaxadiol (50%)	(Bovine serum)	_	0.222 ^e^	_ ^f^	Han et al., (2016) [168]
WMM	Spray drying	Griseofulvin (–)	(HPC SL, Docusate sodium, Mannitol)	Dried powders were dispersed in water and simulated gastric fluid (pH 1.2) using USP II	0.205 ^e^	0.200–0.300 ^e,f^	Shah et al., (2016) [169]
LASP–Ultrasonication	Spray drying	Cefixime trihydrate (–)	(PVP K30, Lactose monohydrate), Mannitol, Sorbitol	Excess amount of dried powder was dispersed in 2 mL distilled water and sonicated for short time	0.266 ^e^	~0.300 ^e,f^	Alaei et al., (2016) [170]
WMM	Wet film casting–drying	Naproxen (–) Anthraquinone (–)	(HPMC, Glycerol, PVP VA64, SDS)	4 cm^2^ film was dispersed in 0.9 mL liquid (DI water, tap water and saliva substitute) for 1–15 min and further diluted with DI water	0.270 0.273	0.280–0.315 ^g^ 0.340–0.420 ^g^	Steiner et al., (2016) [171]
WMM	Fluid bed coating/drying	Itraconazole (14.8%) Fenofibrate (13.6%)	(HPMC, SDS), Hydrophilic silica), GranuLac^®^ 200 (core), PrismaLac^®^ 40 (core)	1 g of dried products were dispersed in 30 mL of 7.2 and 2.88 mg/mL SDS and stirred at 110 rpm for 2 min	0.172 0.171	0.490 ^f^ 0.290 ^f^	Azad et al., (2016) [91]
LASP–Ultrasonication	Freeze drying	Efavirenz (–)	(Poloxamer 407, Soya lecithin, Mannitol)	_	~0.184 ^e^	_ ^f^	Taneja et al., (2016) [172]
Acid-base neutralization–HPH	Freeze drying	Herpetospermum caudigerum lignans (–)	(SDS, PVP K30, Mannitol), Poloxamer 188, Tween 80, HPMC, PVA, Lecithin	Dried products were dispersed in DI water and shaken for 1 min	0.243 ^e^	0.286 ^e,f^	Gang et al., (2016) [173]
HPH	Spray drying	Andrographolide (–)	(HPMC E15/MCCS, Lactose), RH 40, TPGS, PVP K30, Tween 80, Sucrose, Trehalose, Mannitol, Sorbitol	Malvern Mastersizer RDI = mean redispersion size/nanosuspension size ×100	0.500–0.900	~100% ^g^	Xie et al., (2016) [174]
	Freeze drying	Andrographolide (–)	(HPMC E15, Tween 80, Sucrose/Trehalose/Sorbitol), RH 40, TPGS, MCCS, PVP K30, Mannitol, Lactose		0.500–0.900	~100% ^g^	
LASP–Ultrasonication	Freeze drying	Cinnarizine (9.53%)	(PVA)	_	0.621 ^e^	_ ^f^	Mishra et al., (2016) [175]
HPH	Freeze drying	Ritonavir (–)	(HPMC 3, SDS, Mannitol), PVP K30	_	0.562 ^e^	_ ^f^	Karakucuk et al., (2016) [176]
WMM	Freeze drying	BI XX (–)	(Mannitol, Arginine), Polysorbate 80, PEG 400, Proline, Benzalkonium chloride	2 mL DI water was added to the dried product and shaken manually	0.192	~0.250 ^g^	Frank and Boeck (2016) [177]
LASP–Ultrasonication	Wet film casting–drying	Lercanidipine HCl hemihydrate (3.06 mg)	(TPGS 1000, Hypromellose E15), Hypromellose E5, PVA, PEG 400, Sodium alginate, MC, HPC, HPMC E5	4 cm^2^ films were placed in 10 mL DI water and stirred for 10 min using magnetic stirrer	~0.277 ^e^	~0.240 ^e,f^	Chonkar et al., (2016) [178]
WMM	Freeze drying	Spironolactone (–)	(HPMC E5, Sorbitol), Sodium deoxycholate, Poloxamer 407, Poloxamer 188, Mannitol	Dried powders were dispersed in water by gentle shaking	0.374 ^e^	0.399 ^e,f^	Mu et al., (2016) [179]
WMM	Nanoextrusion	Griseofulvin (24.1%)	(HPC SL, SDS), Soluplus	_	0.154	_ ^f^	Li et al., (2017) [78]
WMM	Spray drying	Aprepitant (–)	(Pharmacoat 603, SDS, sucrose), HPMC E15, HPC SSL, PVP K30, TPGS 1000, Poloxamer P188, Mannitol	Dried powders were dispersed in few milliliters of distilled water and shaken manually for 30 s	0.395 ^e^	0.420 ^e,g^	Toziopoulou et al., (2017) [180]
	Freeze drying	Aprepitant (–)	(Pharmacoat 603, SDS, Mannitol)		0.395 ^e^	0.395 ^e,g^	
LASP–HPH	Freeze drying	P2X7 receptor antagonist (–)	(Poloxamer 188, Mannitol), HPMC, SDS	_	0.245 ^e^	_ ^f^	Zhang et al., (2017) [181]
WMM	Freeze drying	Nisoldipine (–)	(PVP K30, SDS, Trehalose), HPMC E5	_	0.240 ^e^	_ ^f^	Fu et al., (2017) [113]
WMM	Freeze drying	Dexamethasone (–) Tacrolimus (–)	(Poloxamer 407) (Poloxamer 407)	Malvern Zetasizer^®^ Nano ZS90	0.403 ^e^0.511 ^e^	0.300–0.700 ^e,g^ 0.300–0.800 ^e,g^	Colombo et al., (2017) [182]
WMM	Electrospraying–Freeze drying	Darunavir (–)	(Tween 20, SLS, Eudragit L100, Mannitol), Poloxamer 338, 188, HPMC, Vitamin E TPGS 400 and 1000, Tween 80	_	0.295 ^e^	_ ^f^	Nguyen et al., (2017) [183]
LASP–Ultrasonication	Freeze drying	Efavirenz (–)	(HPMC E5, SLS), HPC ELF, Poloxamer 188 and 407, PVP K30	_	0.252 ^e^	_ ^f^	Sartori et al., (2017) [184]
WMM	Spray drying	Allisartan Isoproxil (61.7%)	(PVP K30, Mannitol, SDS), Pluronic F127 and F68, Tween 80, HPMC E5, HPMC E50, HPC, PEG 4000 and 6000, Glucose, Sucrose, Maltose, Sorbitol	Dried powders were dispersed in water at a drug concentration of 2.5% (*w*/*v*) and diluted to 900-fold with water	~0.300 ^e^	~0.304 ^e,f^	Hou et al., (2017) [185]
HPH	Freeze drying	Andrographolide (–)	(Glycyrrhizin, Trehalose), Poloxamer 188, Tween 80, TPGS, Sucrose, Lactose	Malvern Mastersizer	0.487	0.491 ^f^	Chen et al., (2017) [186]
WMM	Wet film casting–drying	Griseofulvin (50%)	(HPMC E15LV, SDS, Glycerin), HPMC 4M	~0.7 cm^2^ circular films were dispersed in DI water and vortexed at 1500 rpm for 3–5 min	0.150	<0.200 ^f^	Krull et al., (2017) [83]
WMM	Wet film casting–drying	Anthraquinone (–)	(HPMC, Glycerol), HPC, Kollidon VA64, SDS	4 cm^2^ film dispersed in 0.9 mL distilled water for 5 min	0.302	0.337 ^g^	Steiner et al., (2017) [187]
HPH	Spray drying	Andrographolide (–)	(TPGS, PVP K30, MCC, Lactose), PVPP	Malvern Mastersizer RDI = mean redispersion size/nanosuspension size ×100	~0.514	101.5% ^f^	Xu et al., (2017) [188]
LASP–HPH	Freeze drying	10-hydroxycamptothecin (94.9%) Camptothecin (91.2%) 7-ethyl-10-hydroxycamptothecin (90.1%)	(mPEG1000-HCPT)	Dried products were reconstituted with water	~0.093 ^e^ ~0.121 ^e^ 0.133 ^e^	~0.094 ^e,f^ 0.137 ^e,g^ 0.133 ^e,g^	Yang et al., (2017) [189]
Ultrasonication–HPH	Freeze drying	Meloxicam (–)	(Poloxamer 188, Mannitol), PVP K25, PEG 4000	Dried products corresponding 0.5 mL suspension were diluted to 15 mL with purified water and sonicated for 60 s	~0.463 ^e^	~0.501 ^e,f^	Iurian et al., (2017) [190]
LASP–Ultrasonication	Wet film casting–drying	Lutein (0.23 mg/cm^2^)	(SPC, SDS, HPMC, PEG 400, Cremophor EL), TPGS, PVP K30, Poloxamer 188, PVA	A piece of film was dispersed in 5 mL water and sonicated for 3 min	0.220 ^e^	~0.378 ^e,f^	Liu et al., (2017) [191]
WMM	Fluid bed granulation/drying	Lurasidone hydrochloride (–)	(HPMC E3, Polysorbate 80, Mannitol, MCC), HPMC E5, Poloxamer 188, SLS, Span 20, Labrasol	_	0.245 ^e^	_ ^f^	Kumar et al., (2017) [192]
WMM	Spray drying	Mefenamic acid I (–) Mefenamic acid II (–)	(HPC SSL, Lactose)	_	0.188 0.189	_ ^f^_ ^f^	Konnerth et al., (2017) [193]
LASP–Ultrasonication	Freeze drying	Carvedilol (–)	(SDS), Whey protein isolate, Poloxamer 188	1 mg sample was dispersed in 3 mL of water and shaken manually for 1 min	~0.225 ^e^	0.227 ^e,f^	Geng et al., (2017) [194]

^a^ BCN-Baicalin; CCS: Carboxymethyl cellulose sodium; CMS-Na: sodium carboxymethyl starch; CP: Crospovidone; Cremophor^®^ RH 40: Polyoxyethylene hydrogenated castor oil; Dowfax 2A1: alkyldiphenyloxide disulfonate; GG: Guar gum; HPBCD: hydroxypropyl β-cyclodextrin; HPMC: Hydroxypropylmethylcellulose; MC: Methylcellulose; MCC: Microcrystalline cellulose; MCCS: Microcrystalline cellulose and carboxymethyl cellulose sodium; MRES: Maleic rosin-polyoxypropylene-polyoxyethylene ether sulfonate; MMT: Montmorillonite (clay); NGN-Naringenin; OCA-Oleanolic acid; PEG-PLA: Polyethyleneglycol-polylactide; PEG4000: Polyethylene glycol; PVP: Polyvinylpyrrolidone; mPEG1000-HCPT: Polyethylene glycol (PEG)ylated 10-hydroxycamptothecin; RCN-Rubescensin; RPN-Rutacarpine; RVL-Resveratrol; SDS: Sodium dodecyl sulfate; SLS: Sodium lauryl sulfate; SPC: Soy phosphatidylcholine; SSG: Sodium starch glycolate; TPGS 1000: D-α-tocopheryl polyethylene glycol 1000 succinate; XG: Xanthan gum; RDI: Redispersibility index. ^b^ The dispersants in the parenthesis refer to the particular nanocomposite formulation that led to the finest drug particle sizes upon redispersion, or that led to the fastest dissolution if redispersion was not performed. ^c^ Unless otherwise indicated, all particle sizes refer to the median size (*d*_50_) of the nanosuspension (before redispersion) and the redispersed suspension (after redispersion) for the particular nanocomposite formulation that led to the finest drug particle sizes. If redispersion was not performed, “before redispersion” particle size refers to the particle size of the nanosuspension with the finest particle size. If multiple redispersion methods were used, the specific redispersion method used for the reported particle sizes is underlined. ^d^ Volume-mean diameter. ^e^ Cumulant size was measured by dynamic light scattering. ^f^ Fastest dissolving formulation. ^g^ Dissolution testing was not performed.

**Table 3:** Classification of dispersants used in drug-laden nanocomposites.

Class	Examples	Possible Mechanisms of Action	References
Soluble polymers	HPC, HPMC, PVP, Soluplus, PEG, PVA, Sodium CMC, methyl cellulose, PEG-PLA, Sodium alginate	Steric stabilizer in nanosuspension, enhanced drug wettability in nanocomposites, primary matrix/film former that prevents aggregation during drying and facilitates erosion/disintegration of nanocomposites via dissolution.	Lee et al., 2003 [33], Li et al., 2016 [23], Chin et al. [55]
Surfactants	Alpha tocopherol succinate, SDS, TPGS, Poloxamer 338, Pluronic F127, Pluronic F68, Span 20/60, Tween 20/60, Dowfax 2A1, Soy lecithin, Docussate sodium, Cremophor, Sodium deoxycholate	Steric or electrostatic stabilizer depending on its charge, wetting agent, enhanced drug wettability in nanocomposites	Bonda et al., 2016 [163], Li et al., 2016 [23], Chin et al., [55]
Other water-soluble dispersants (WSD)	Sucrose, Lactose, Trehalose, Glucose, Maltose, Maltodextrin, Ficoll PM70, HPβ-cyclodextrin, L-Leucine, Mannitol, Arginine, sodium carboxymethyl starch (CMS-Na)	Secondary matrix former that prevents aggregation during drying, facilitates erosion/disintegration of nanocomposite particles via dissolution, and act as cryoprotectant in freeze drying	Abdelwahed et al., 2006 [197], Kesisoglou et al. [28], Chin et al. [55]
Water-insoluble dispersants (WID)	Microcrystalline cellulose, anhydrous dicalcium phosphate, colloidal fumed silica, montmorillonite	Secondary matrix former that prevents aggregation during drying and facilitates erosion/disintegration of nanocomposites	Eerdenbrugh et al., 2008 [59]
Crosslinked polymers (CLP)	SSG, CCS, CP	Swellable dispersant that facilitates erosion/disintegration of nanocomposite particles via swelling-induced breakage/erosion of nanocomposite matrix	Bhakay et al., 2014 [8], Azad et al., 2015 [27]
